# The long non-coding RNA Meg3 mediates imprinted gene expression during stem cell differentiation

**DOI:** 10.1093/nar/gkae247

**Published:** 2024-04-13

**Authors:** Sabina Farhadova, Amani Ghousein, François Charon, Caroline Surcis, Melisa Gomez-Velazques, Clara Roidor, Flavio Di Michele, Maud Borensztein, Albertina De Sario, Cyril Esnault, Daan Noordermeer, Benoit Moindrot, Robert Feil

**Affiliations:** Institute of Molecular Genetics of Montpellier (IGMM), Centre National de Recherche Scientifique (CNRS), 34090 Montpellier, France; University of Montpellier, 34090 Montpellier, France; Genetic Resources Research Institute, Azerbaijan National Academy of Sciences (ANAS), AZ1106 Baku, Azerbaijan; Institute of Molecular Genetics of Montpellier (IGMM), Centre National de Recherche Scientifique (CNRS), 34090 Montpellier, France; University of Montpellier, 34090 Montpellier, France; Université Paris-Saclay, CEA, CNRS, Institute for Integrative Biology of the Cell (I2BC), 91190 Gif-sur-Yvette, France; Institute of Molecular Genetics of Montpellier (IGMM), Centre National de Recherche Scientifique (CNRS), 34090 Montpellier, France; Institute of Molecular Genetics of Montpellier (IGMM), Centre National de Recherche Scientifique (CNRS), 34090 Montpellier, France; University of Montpellier, 34090 Montpellier, France; Institute of Molecular Genetics of Montpellier (IGMM), Centre National de Recherche Scientifique (CNRS), 34090 Montpellier, France; University of Montpellier, 34090 Montpellier, France; Institute of Molecular Genetics of Montpellier (IGMM), Centre National de Recherche Scientifique (CNRS), 34090 Montpellier, France; University of Montpellier, 34090 Montpellier, France; Institute of Molecular Genetics of Montpellier (IGMM), Centre National de Recherche Scientifique (CNRS), 34090 Montpellier, France; University of Montpellier, 34090 Montpellier, France; University of Montpellier, 34090 Montpellier, France; PhyMedExp, Institut National de la Santé et de la Recherche Médicale (INSERM), CNRS, 34295 Montpellier, France; Institute of Molecular Genetics of Montpellier (IGMM), Centre National de Recherche Scientifique (CNRS), 34090 Montpellier, France; University of Montpellier, 34090 Montpellier, France; Université Paris-Saclay, CEA, CNRS, Institute for Integrative Biology of the Cell (I2BC), 91190 Gif-sur-Yvette, France; Université Paris-Saclay, CEA, CNRS, Institute for Integrative Biology of the Cell (I2BC), 91190 Gif-sur-Yvette, France; Institute of Molecular Genetics of Montpellier (IGMM), Centre National de Recherche Scientifique (CNRS), 34090 Montpellier, France; University of Montpellier, 34090 Montpellier, France

## Abstract

The imprinted *Dlk1-Dio3* domain comprises the developmental genes *Dlk1* and *Rtl1*, which are silenced on the maternal chromosome in different cell types. On this parental chromosome, the domain's imprinting control region activates a polycistron that produces the lncRNA Meg3 and many miRNAs (*Mirg*) and C/D-box snoRNAs (*Rian*). Although Meg3 lncRNA is nuclear and associates with the maternal chromosome, it is unknown whether it controls gene repression in *cis*. We created mouse embryonic stem cells (mESCs) that carry an ectopic poly(A) signal, reducing RNA levels along the polycistron, and generated *Rian*^−/−^ mESCs as well. Upon ESC differentiation, we found that Meg3 lncRNA (but not *Rian*) is required for *Dlk1* repression on the maternal chromosome. Biallelic *Meg3* expression acquired through CRISPR-mediated demethylation of the paternal *Meg3* promoter led to biallelic *Dlk1* repression, and to loss of *Rtl1* expression. lncRNA expression also correlated with DNA hypomethylation and CTCF binding at the 5′-side of *Meg3*. Using Capture Hi-C, we found that this creates a Topologically Associating Domain (TAD) organization that brings *Meg3* close to *Dlk1* on the maternal chromosome. The requirement of Meg3 for gene repression and TAD structure may explain how aberrant *MEG3* expression at the human *DLK1-DIO3* locus associates with imprinting disorders.

## Graphical Abstract

**Graphical Abstract:**
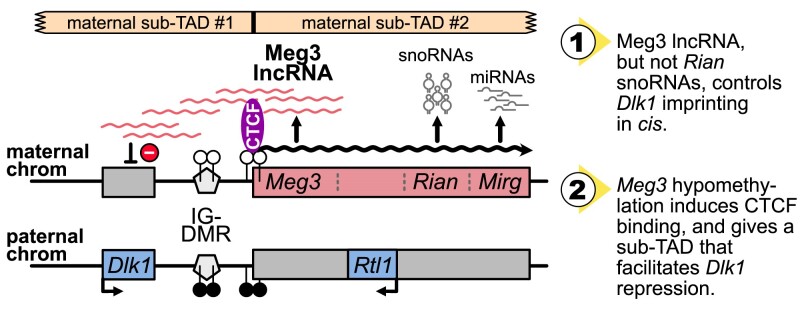

## Introduction

Genomic imprinting is an epigenetic phenomenon that plays diverse roles in development, metabolism and behavior. It mediates the mono-allelic expression of ∼150 protein-coding genes—strictly dependent on their parental origin—to critically control their expression dosage (1). In addition, hundreds of regulatory non-coding RNAs (ncRNAs) are also imprinted, whose functions often remain poorly characterized. Most imprinted domains, for instance, express at least one long non-coding RNA (lncRNA) (2–5).

**Figure 1.**
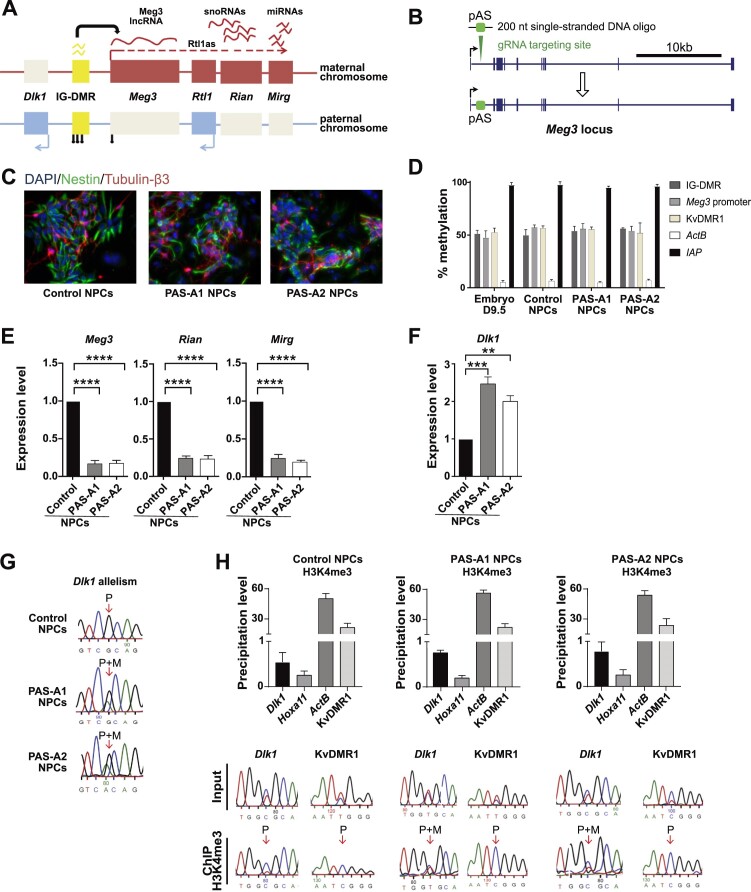
pAS insertion into *Meg3*-intron-1 abrogates *Dlk1* imprinted expression in NPCs. (**A**) Schematic presentation of the imprinted *Dlk1-Dio3* domain, at which the *Meg3* promoter drives the expression of the *Meg3-Rian-Mirg* ncRNA polycistron. Rectangles represent genes with their maternal- (red) or paternal (blue)-specific expression. The yellow rectangle depicts the ICR, called IG-DMR (6), which is methylated (lollipops) on the paternally inherited chromosome. Primary transcripts from *Meg3-Rian-Mirg* polycistron are represented by a dashed line; wave-shaped lines indicate processed ncRNAs. (**B**) CRISPR insertion of a 49-bp synthetic p(A) signal (‘pAS’) into *Meg3* intron-1, using a single-stranded DNA oligo with homology arms (200 bp in total). Blue rectangles depict the exons of *Meg3*. (**C**) Immunofluorescence (IF) staining of Nestin (green) and Tubulin-β3 (red) with DAPI counter-staining (blue) in hybrid mESC-derived NPCs at d12 of neural differentiation. (**D**) DNA methylation status in hybrid mESC-derived NPCs (control, PAS-A1 and PAS-A2) and in E9.5 embryos, determined by methylation-sensitive qPCR. Controls: KvDMR1 (a maternally methylated ICR on chromosome 7), *ActB* promoter (low methylation) and *IAP* elements (high methylation). Bars represent means ± SD from three experiments. (**E**) Levels of Meg3, Rian and Mirg spliced RNAs assessed by RT-qPCR on total-RNAs relative to housekeeping genes (*β-actin* and *Gapdh*) in control, PAS-A1 and PAS-A2 NPCs. Bars represent means ± SD from three experiments (**** *P*< 0.0001). (**F**) *Dlk1* mRNA amounts relative to housekeeping genes (*β-actin* and *Gapdh*) in control, PAS-A1 and PAS-A2 NPCs. Bars represent means ± SD from three independent experiments (**P< 0.01, *** *P*< 0.001). (**G**) Sanger sequencing-based assessment of *Dlk1* allelism in control, PAS-A1 and PAS-A2 NPCs. The arrow indicates the SNP used to distinguish the maternal (M) and paternal (P) alleles. (**H**) H3K4me3 ChIP on control, PAS-A1 and PAS-A2 NPCs. Percentile precipitation levels were determined by qPCR at the *Dlk1* promoter. *Hoxa11* and *ActB* are negative and positive control regions, respectively. The KvDMR1, a control ICR, is transcriptionally active on the paternal chromosome only. Bottom: Sanger sequencing profiles indicate the allele-specificity of H3K4me3 at *Dlk1* and the KvDMR1.

The imprinted *Dlk1-Dio3* locus on mouse chromosome 12qF1 (Figure 1A) is controlled by an intergenic Imprinting Control Region (ICR)—called the intergenic Differentially Methylated Region (IG-DMR)—which is methylated on the paternal chromosome (6). The unmethylated maternal copy of this ICR acts as an enhancer that activates the nearby *Meg3* promoter, thereby driving the expression of a 220-kb polycistron (7–10). This complex transcription unit generates the lncRNA Meg3 (‘Maternally expressed gene 3′, also called Gtl2) and many small RNAs, including miRNAs of the *Mirg* (‘microRNA containing gene’) cluster and C/D-box snoRNAs of the *Rian* (‘RNA imprinted and accumulated in the nucleus’) locus (referred to as *MEG8* in humans) (8,11–13).

On the paternal chromosome, the *Dlk1-Dio3* domain predominantly expresses two protein-coding genes with a distinct developmental dynamics (8,14,15). These are the *Dlk1* gene (the atypical Notch-ligand-encoding ‘Delta-like homologue-1’) and the retrotransposon-derived gene *Rtl1* (‘Retrotransposon gag-like 1’), which expresses a gag-like protein (14,16). *Rtl1* overlaps the *Meg3–Rian–Mirg* polycistron, which expresses on the maternal chromosome sequences antisense to Rtl1 RNA (Figure 1A). A third protein-coding gene located downstream of the *Meg3* ncRNA polycistron, thyroxine deiodinase type III (*Dio3*), shows a weak paternal expression bias (6,17–19).

While there is no evidence that the IG-DMR acts as an enhancer for the *Dlk1* and *Rtl1* genes on the paternal chromosome, the allelic repression of these genes on the maternal chromosome does require the maternal ICR (6,20). Specifically, the 3′ part of the ICR is critically required for its repressive effects on *Dlk1* (7). Since this part of the IG-DMR displays enhancer features, and activates in *cis* the *Meg3* promoter that drives the expression of the polycistron (8,9,21), it has been hypothesized that the expression of the *Meg3-Rian-Mirg* polycistron could be responsible for the in-*cis* repression of *Dlk1* during development (7,8,10,14). Indeed, gene targeting studies in the mouse have shown that loss of *Meg3–Rian–Mirg* expression correlates with a loss of *Dlk1* imprinting (i.e. biallelic expression) (6,7,10,20,22,23).

In humans, epimutations and microdeletions that affect the IG-DMR or the *MEG3* promoter are observed in two congenital imprinting disorders (IDs), Kagami-Ogata Syndrome (KOS14, OMIM 608149) and Temple Syndrome (TS14, OMIM 616222) (24–27). KOS14 and TS14 have in common that the activity of the polycistron is either fully ablated, or becomes biallelic, respectively. Similar as in mice, this evokes a putative *cis* role of the *MEG3-RIAN (MEG8)-MIRG* polycistron in *DLK1* gene expression dosage. In patients with these IDs, however, methylation changes are often mosaic and can involve multiple loci, which complicates interpretations about individual genes (28,29).

In support of a putative repressive role of Meg3 RNA, fluorescence *in situ* hybridization (FISH) studies on mESCs have shown that this lncRNA is retained in part on the imprinted domain and ‘overlaps’ *Dlk1* on the maternal chromosome (22). Concordantly, Chromosome Conformation Capture (3C) based studies indicate that the *Meg3* promoter is positioned in close proximity to the *Dlk1* gene on the maternal chromosome, through the formation of an allele-specific sub-TAD (Topologically Associating Domain) (7,30). The maternal-specific boundary of this sub-TAD, formed by a non-methylated binding site for the CTCF insulator protein within intron-1 of *Meg3*, is critical for *Dlk1* repression (7,30). This allele-specific 3D-organization might thus provide a framework for (or result from) the focal accumulation of Meg3 lncRNA. In both cases, it remains unclear whether *Meg3* proximity to the *Dlk1* locus is functionally relevant for its imprinting, and whether Meg3 *cis*-retention is required. As such, the precise role of Meg3 lncRNA, and that of the other ncRNAs of the polycistron, in imprinted gene expression remains unclear.

To address these questions, we engineered genetic modifications to the *Dlk1-Dio3* domain in F1-hybrid mouse embryonic stem cells (mESC, C57BL/6J x JF1) that faithfully recapitulate imprinted gene expression upon *in vitro* differentiation (8,22,31). Multiple cell lines with decreased expression along the *Meg3–Rian–Mirg* polycistron were generated, and the effects of *Rian* deletion were explored as well. In addition, we induced biallelic *Meg3* expression by CRISPR-mediated demethylation of the (normally silenced) paternal promoter, to assess the precise effects on imprinted gene expression and chromatin structure at the *Dlk1-Dio3* domain. Our findings highlight the importance of Meg3 lncRNA—and not of *Rian*—in differential chromatin 3D structure and the *in-cis* repression of *Dlk1*. This essential role helps to understand complex imprinting-related disorders in humans, such as TS14 and KOS14, in which patients show aberrant *MEG3* expression.

## Materials and methods

### mESC derivation, culture and differentiation

mESCs were derived under serum-free conditions in ESGRO 2i medium (Sigma-Aldrich, #SF016-200) from blastocysts that were hybrid between *M. m. domesticus* strain C57BL/6 J and *M. m. molossinus* strain JF1 (22,32). mESCs were maintained on gelatin-coated dishes in synthetic ESGRO 1i medium (Sigma-Aldrich, #SF001-500P) without serum, systematically supplemented with l-ascorbic acid (50 ug/ml) to prevent aberrant DNA methylation (33,34). For the detachment of cells, Accutase solution (Millipore, #SCR005) was used. mESCs were differentiated into neural progenitor (NPCs) cells for 12 days in the presence of the kinase inhibitor DMH1 in substitution of cyclopamine, but otherwise following a previously published protocol (35,36). mESC differentiation into cardiomyocytes (CMCs) was similar as reported by others before (37). Briefly, control or gene-edited mESCs were cultured for 2 days and were dissociated, to then form aggregates in differentiation medium [DMEM-Glutamax (4.5 g/l d-glucose + pyruvate; Gibco, #31966-021) supplemented with 20% fetal bovine serum (Gibco, #10500-064), 0.1 mM non-essential amino acids (Gibco, #11140-035), 100 μM β-ME (Gibco, #31350-010)] using the hanging drop method. Hanging drops comprised between 700 and 1000 cells, and were cultured for 3 days in 20 μl differentiation medium. The obtained embryoid bodies (EBs) were plated individually onto 0.1% gelatin-coated 24- or 96-well plates, or on microscope cover slips placed within culture dishes, in differentiation medium that was changed every 2 days. Cultures were analysed following 12 days of differentiation when most of the outgrowing EBs comprise beating cardiomyocytes. Cardiomyocyte differentiation was monitored by assessing the developmental marker genes *Tbx5, Gata4, Mesp1* and *Nkx2.5* (37) and by immuno-fluorescence staining of cardiac Troponin-T.

### CRISPR-Cas9 mediated pAS insertion and *Rian* deletion

gRNAs were designed using CRISPR Design tool (http://crispr.mit.edu/), and presented in Supplementary Table S1. gRNAs were synthesized to be flanked with *Bbs*I sticky ends and cloned into pSpCas9(BB)-2A-GFP plasmid (Addgene, #48138). Constructs were transfected into mESCs using Amaxa nucleofector (Lonza, #VPH-1001). GFP-positive cells were sorted by flow cytometry (FACS Aria, Becton Dickinson) after 48 h of transfection, and plated as single cells in 96 well plates. As a control experiment, a non-specific, scrambled gRNA was used to generate control mESC lines that had undergone the same manipulations. Cell lines were established from single colonies. For pAS insertion, a plasmid carrying one gRNA was transfected into mESCs in addition to a homology repair sequence that ensured a proper pAS insertion at the desired location. Colony PCR testing identified cell lines harboring the pAS insertion, and two lines were selected for further study: PAS-A1 (collection name: BJ-PAS-A3) and PAS-A2 (collection name BJ-PAS-A19). A CRISPR-Cas9 NHEJ-mediated deletion genome-editing approach was used to delete the *Rian* snoRNA cluster, using two gRNAs. RT qPCR and RNA-seq confirmed that the entire *Rian* cluster was deleted in the selected ESC clones: *Rian*^−/−^[1] (collection name BJ-Rian 59) and *Rian*^−/−^[2] (collection name BJ-Rian 75).

### CRISPR-dCas9-TET1-mediated DNA demethylation

gRNAs are described in Supplementary Table S1. The gRNA for the *H19* ICR was used by others before (38). gRNAs for the *Meg3* DMR were designed about 1 kb upstream of exon-1 in the annotated promoter regions. sgRNAs were annealed and cloned into the pPlatTet-gRNA2 plasmid (Addgene, #82559; a kind gift from Dr Izuho Hatada) after digestion by *Afl*II and Gibson assembly (38). Plasmids expressing gRNA were electroporated into mESCs using Amaxa nucleofector (Lonza, #VPH-1001). GFP-positive cells were selected 48 h post-electroporation by flow cytometry (FACS Aria, Becton Dickinson), and single cells were seeded onto 96-well plates. After 10–12 days of culture, individually picked colonies were grown in 6-well plates to derive ESC clones. Their methylation levels were determined by methylation-sensitive enzymatic digestion.

### Immunofluorescence staining

Immunofluorescence staining of cells was performed as reported before (8). The used primary antisera were directed against Nestin (Biolegend, #839801), Tubulin-B3 (Biolegend, #801201) and Troponin-T (Invitrogen, #13-11 MAS-12960). Secondary antibodies used: goat anti-mouse Alexa fluor 488 (Thermo-Fisher, #A-11011) or goat anti-rabbit Alexa Fluor 594 (Thermo-Fisher, #A-11012).

### RNA and DNA FISH

RNA-FISH was performed as in Chaumeil *et al.* 2008 (39), on cells grown on gelatin-coated coverslips and fixed with 4% paraformaldehyde for 10 min at room temperature. Cells were permeabilized in 0.5% v/v Triton X-100 in 1× PBS (10 min, on ice), rinsed in 2× PBS (5 min, RT) and store at 4°C in 70% ethanol. RNA FISH against total Meg3 RNA was with a cDNA probe (Openbiosystems, #6831921), and with a probe comprising intron1 + intron8 sequences (Chr.12, mm10: 109541150–109542018, 109554267–109555402, 109556110–109557125 and 109555037–109557125). For simultaneous RNA- and DNA-FISH, cells were re-permeabilised in 0.5% v/v Triton X-100 in 1× PBS (20 min, RT), rinsed in 2× PBS (5 min, RT) and 70% ethanol (5 min, RT). Cells were dehydrated for 3 min subsequently, with respectively 80%, 95% and 100% ethanol. Before hybridization, cells were denatured in 50% formamide, 2× SSC (pH 7.2) for 30 min in water bath at 80°C; then, washed twice in 2× SSC on ice. We used a *Dlk1-*comprising fosmid (WIBR1-1116K16; Chr12: 110674199–110709802, BACPAC Resources Center) and Meg3 cDNA as probes, labelled with fluorescent nucleotides by nick translation using 1 μg of DNA per 50 μl of reaction following manufacturer's instructions (Abbott, #07J00-001). Per coverslip, approximately 0.1 μg of probe was ethanol-precipitated together with 10 μg of salmon sperm DNA and 1ug of cot-1 DNA, air-dried and resuspended in 12.5 μl of formamide. *Dlk1* fosmid and Meg3 cDNA probes were then mixed in 12.5 μl hybridization cocktail consisting of 2× hybridization mixture (4× SSCT, 20% w/v dextran sulfate, 2 mg/ml BSA, 40 mM Vanadyl Ribonucleoside Complex). Cells were incubated in hybridization cocktail, overnight at 40°C in a dark humidified chamber. The next day, cells were washed three times with 50% formamide, 2× SSC at 42°C for 5 min, followed by three times 5 min with 2× SSC at 42°C, and stained with DAPI and mounted using Vectashield antifade 18 mounting medium (VectorLabs, #H-1000). Images were acquired on a laser scanning confocal microscope (LSM980 Airyscan 8Y, Zeiss) with 63× NA1.4 Plan-Apochromat objective (Zeiss). z stacks of 0.2-μm slices were visualized and analysed using OMERO Open Microscopy Environment (OME) and ImageJ tools. For foci co-localization measurements, we used JACoP plugin in ImageJ.

### RNA expression analyses

Total RNA was extracted using RNeasy-plus mini kit (Qiagen) and transcribed into cDNA using random hexamers and Superscript III reverse transcriptase (Thermo Fisher). In subsequent RT-qPCR, measured expression levels were normalized to the geometric mean of two housekeeping genes (*Actb* and *Gapdh)* as reported before (22). For the analysis of *Rtl1* expression levels, strand-specific oligonucleotides were used for cDNA synthesis in multiple experiments, followed by *Rtl1*-specific amplification; a one-way Anova test was used for statistical comparison (40). Primer sequences are presented in Supplementary Table S2.

For sequencing of nuclear RNAs, nuclei were purified by incubating 15.10^6^ resuspended cells in 4 ml of Buffer I (10 mM Tris–HCl pH 7.5, 10 mM NaCl, 2.5 mM MgCl_2_, 0.5% IGEPAL CA-630) on ice for 5 min. Next, we carefully underlaid 1 ml of Buffer II (10 mM Tris–HCl pH 7.5, 10 mM NaCl, 2.5 mM MgCl_2_, 0.5% IGEPAL CA-630, 10% sucrose) and harvested the nuclear fraction at 1400 rcf for 5 min at 4°C. Nuclear RNAs were isolated using RNeasy Plus Mini Kit (Qiagen, #74136); they were quantified by Qubit and quality was assessed using the RNA Assay kit (Agilent RNA 6000 Pico reagents, #1567-1513) with Bioanalyzer 2100 (Agilent Technologies, USA). Putative residual genomic DNA was digested using Amplification-Grade DNase I (AMPD1, Sigma) and spike-in ERCC (Thermofisher, # 4456740) was added following manufacturer instructions before library preparation of RNA samples. For library preparation we used the True-seq stranded total RNA library prep gold kit (Illumina, #220599) following the manufacturer's instructions (including ribo-depletion). Sequencing datasets were aligned to the mouse genome (mm39) using TopHat2. Aligned reads were treated using an in-house developed PASHA (version 0.99.21) R (version 3.3.1) pipeline to generate wiggle files. Read counts within the ERCC spike-in genome were determined by HTSeq-count (version 0.6.1p1) and used to normalize the wiggle files for visualization as reads per million reads of the spiked-in genome.

### ChIP and CUT&RUN

Chromatin immuno-precipitation (ChIP) was performed as described before (22). Briefly, cells were fixed in 1% (v/v) formaldehyde for 10 min at RT, followed by 1 × 10 cycles (30 s on/30 s off) of sonication in a BioRuptor Pico sonicator. Around 15 million cells were used for each IP. The antisera used were: 5 μg of anti-H3K4me3 (Active Motif, #39155), 10 μg of anti-CTCF (Merck Millipore, #07-729). Precipitated DNA was purified using the ‘ChIP DNA clean and concentrator kit’ (Zymo Research, #D5205) and quantified by qPCR. PCR products were run on a 1% agarose gel, excised, and column-purified (Macherey-Nagel, #740609.10). The allelism was determined by Sanger sequencing of qPCR product across SNPs. Primer sequences are given in Supplementary Table S3. CUT&RUN (Cleavage Under Targets and Release Using Nuclease) experiments were as described before (Roidor, Syx *et al.* 2023 BiorXiv; doi: https://doi.org/10.1101/2023.04.25.532252). Briefly, for each sample 200 000 cells were pelleted. Nuclear Extraction Buffer (20 mM HEPES–KOH, 10 mM KCl, 0.5 mM spermidine, 0.1% Triton X-100, 20% glycerol, complete EDTA-free protease inhibitor cocktail) was gently added to the pellet, with incubation on ice for 5 min. Nuclei were then re-suspended in Nuclear Extraction Buffer and stored at –80°C for up to several weeks. The antisera used for CUT&RUN were anti-H3K27me3 (Cell signalling 36B11#9733) and anti-IgG (Sigma, #I5006), followed by addition of Protein-A-MNase fusion protein (a kind gift of Dr D. Helmlinger), to 200 ng/ul. The MNAse-digested DNA (fragments) diffused out of the cells was precipitated after 30 min, and loci of interest were quantified by qPCR. In addition, PCR products were run on a 1.5% agarose gel, excised, and column-purified (Macherey-Nagel, #740609.50), followed by Sanger sequencing to distinguish the parental alleles. PCR primer sequences are given in Supplementary Table S3.

### DNA methylation analysis

Methylation levels were analysed through digestion with methylation-sensitive restriction endonucleases (*Aci*I, *Hpa*II or *Hha*I), depending on the genomic locus studied, followed by qPCR. Briefly, 1 μg of genomic DNA was digested with *Eco*RI in a 100 μl reaction volume. After 3 h, the reaction volume was divided into two tubes, each containing 40 μl of the initial reaction volume, with one Eppendorf tube without, and a second tube with, addition of methylation-sensitive restriction enzyme. 1 ng of DNA from each tube was used for qPCR. The percentage of methylation for each region was calculated, and the standard curve method was used to quantify values. Values were normalized to the amplification levels of two non-methylated control regions (*Col1a2* and *Col9a2)* (30). Primer sequences are provided in Supplementary Table S4. For Reduced Representation Bisulfite Sequencing (RRBS), 200 ng of genomic DNA was digested with *Msp*I for 5 h, followed by end-repair, A-tailing with Klenow fragment (ThermoScientific, #EPO-421), and ligation to methylated indexed Illumina adapters using T4 DNA ligase (ThermoScientific, # 15224017). Fragments were purified using AMPure XP magnetic beads (Beckman Coulter) (41). Two rounds of bisulfite conversion were then performed using the EpiTect kit (Qiagen, #59104). Final RRBS libraries were PCR-amplified with PfuTurbo Cx hot-start DNA polymerase (Agilent, #600410) as follows: 95°C for 2 min, 14 cycles (95°C for 30 s, 65°C for 30 s, and 72°C for 45 s), 72°C for 7 min. Next, the libraries were purified with AMPure XP magnetic beads, quantified with a Qubit fluorometer (Life Technologies), and verified by Fragment analyzer (Advanced Analytical) and qPCR. Directional libraries were sequenced (100 nt single-end reads) on an Illumina HiSeq2000 at the MGX facility. Reads were processed and analyzed with tools developed by the Babraham Institute, Cambridge, UK (Trim_galore, Bismark and Seqmonk) (42). For pyrosequencing, 1 μg of genomic DNA was treated with sodium bisulfite using the EpiTect kit (Qiagen, #59104). PCR products were amplified with the PyroMark PCR kit (Qiagen, #978703) and purified with Streptavidin Sepharose HP™ (GE Healthcare, #17–5113-01) using a PyroMark Q24 Workstation. Pyrosequencing was done with Gold Q24 reagents (Qiagen, #970802) using a PyroMark Q24. PCR- and sequencing primers are provided in Supplementary Table S5.

### 4C-seq, Capture-C and Capture Hi-C experiments

4C-seq experiments on WT and Meg3-TET-46 mESCs were as described before (30,43). For the IG-DMR viewpoint, only the JF1 (paternal allele) was analyzed. For other viewpoints, both alleles were targeted and reads were assigned to their corresponding allele using a single nucleotide polymorphism (SNP) located four nucleotides 3′ of the forward 4C-seq primers. Downstream analyses were done using the c4ctus pipeline (43), with customized Perl and R scripts to distinguish the parental alleles based on SNPs (scripts available upon request from BM). Primer sequences are given in Supplementary Table S6. The Capture-C strategy used for WT and Meg3-TET-46 mESCs was adapted from the C-TALE protocol (44). Briefly, 3C material was prepared from cross-linked cell pellets as described before (43). DNA was fragmented using a Covaris S220 apparatus (10% Duty Factor, 140W incident Power, 200 cycles per burst for 55 s) and converted into Illumina compatible libraries using separate NEBNext Ultra II modules (NEB, #E6050, #E6053 and #E6056) following the manufacturer's instructions. Three or four aliquots of 200 ng of adaptor ligated libraries were amplified using KAPA HiFi HotStart for 5 cycles. To generate biotinylated probes for enrichment, an equimolar mix of BACs (RP23-75I2, RP23-132J1, RP23-409I23, ordered from Source BioScience) was fragmented by enzymatic digestion (with *Mbo*I) followed by sonication (Covaris S220; 10% duty factor, 150 W incident power, 200 cycles per burst for 180 sec) and biotinylated as described (44). Two rounds of target enrichment were performed, using reagents and instructions from the Twist Hybridization and Wash kit (Twist, #101279) and Twist Universal Blockers (Twist, #100856). For both rounds, the enrichment was performed on a total of 1.5 μg Illumina compatible material, with the post-capture PCR reactions done using KAPA HiFi polymerase (12 PCR cycles). After the second enrichment step, size-selection was by sequential SPRI bead binding (Beckman-Coulter, 0.6× and 0.9× volumes).

For Capture Hi-C on differentiated control CMCs and NPCs, epimutated CMCs and mutant NPCs, a commercially available kit was used. Hi-C material was prepared from 1 million cross-linked cells using the Arima Hi-C+/High Coverage Hi-C kit (Arima Genomics), following the manufacturer's instructions. Sequencing libraries were prepared using the SureSelect XT HS2 DNA System kit (Agilent). Target enrichment was performed using a custom panel targeting the coordinates chr12:108930000–110030000 (Agilent; mm10), following the manufacturer's instructions. For both Capture-C and the Capture Hi-C, the material was sequenced on the Illumina NextSeq 500 (paired-end, 2 × 43) at the High-throughput sequencing facility of I2BC. Reads were processed using the Hi-C pro tool (45) using the JF1-specific SNPs from the Mouse Genome Project REL-1807. Allele-specific matrices, generated at 10-kb resolution, were displayed and analyzed using R. Indicated coordinates are from GRCm38/mm10.

## Results

### Insertion of a poly(A) signal into *Meg3* reduces *Meg3*, *Rian* and *Mirg* expression and attenuates *Dlk1* imprinting

The *Meg3-*promoter-driven expression of the *Meg3–Rian–Mirg* polycistron (Figure 1A) is required to prevent *Dlk1* activation on the maternal chromosome (22). Whether this repressive role is mediated by the *Meg3* promoter, its transcriptional activity, or by one or more of the many ncRNAs produced by this large polycistron (46), is unclear. To address this important question, we used CRISPR-Cas9-mediated recombination to insert a poly(A) signal (pAS) within *Meg3*, with the aim of generating mESC lines with strongly reduced *Meg3–Rian–Mirg* polycistron expression. We used a synthetic pAS of 49-bp in length adapted from the rabbit β*-globin* pAS (47) that had yielded truncated lncRNA transcripts in earlier studies by others (48,49). Experiments were performed on naïve hybrid mESCs and we systematically supplemented the serum-free ESC medium with ascorbic acid, to prevent acquisition of aberrant *de novo* DNA methylation (33,34).

To reduce RNA levels along the entire polycistron, while maintaining the *Meg3* promoter in an active state, we inserted one copy of the synthetic pAS into intron-1 of *Meg3*, 240-bp downstream of the transcription start site (TSS) (Figure 1B). Following electroporation of WT mESCs [of (C57BL/6J x JF1)F1 genotype]—hereafter referred to as ‘BJ’—gene-edited cells were purified by cytometric cell sorting based on Cas9-GFP expression. Following colony formation, pAS insertion was ascertained by PCR amplification followed by DNA sequencing of the obtained PCR products. Two independent clones were selected for further studies—with biallelic and maternal PAS insertion respectively—which we named ‘PAS-A1’ and ‘PAS-A2’ (Supplementary Figure S1A). As a negative control, a non-specific scrambled gRNA was used, to generate suitable control mESCs.

Methylation levels at the IG-DMR and *Meg3* promoter, as assayed using methylation-sensitive enzymatic digestion, remained at about 50%, as expected from the allelic nature of the methylation imprint (Supplementary Figure S1B). Because of the pAS insertion, the expression of *Meg3*, *Rian* and *Mirg* was reduced to about half compared to control mESCs (Supplementary Figure S1C). RNA FISH showed that the percentage of cells with focal lncRNA retention was similar (∼70%) as in the control cells (Supplementary Figure S1D).

To induce neural differentiation, we applied a previously published procedure to generate neural progenitor cells (NPCs) from mESCs (31,35). PAS-A1 and PAS-A2 mESCs readily differentiated into NPCs, with similar efficiency as control BJ mESCs. At day 12 (d12) of differentiation, NPCs displayed axonal outgrowth and showed similar protein expression of Nestin and Tubulin-β3 as NPCs generated from control mESCs (Figure 1C). Similarly, RT-PCR analysis on total-RNA samples confirmed that the neural marker genes *Nestin*, *Fabp7* and *Emx1* were expressed at similar levels in the PAS-A1 and PAS-A2 versus the control NPCs (Supplementary Figure S1E). DNA methylation at the *Meg3* promoter and the IG-DMR remained unaltered upon neural differentiation of the PAS-A1 and PAS-A2 cells (Figure 1D).

In the obtained NPCs, the transcriptional reduction was more pronounced than in mESCs, with the Meg3 (5′ part of the spliced transcript), Rian and Mirg transcript levels reduced by 70–80% (Figure 1E). To determine whether the reduced Meg3 levels included the primary transcript, and the 3′ portion of the gene, we performed additional RT-qPCR amplifications at exon 8 and intron 8. At both these regions, there was a similar reduction (70–80%) in the PAS-A1 and PAS-A2 NPCs (Supplementary Figure S1F).

In control NPCs, substantial parts of the Meg3 RNA were present at high levels in the nucleus, presumably through post-transcriptional accumulation. RNA FISH with a Meg3 cDNA probe revealed multiple accumulation foci in the nucleoplasm of NPCs. This observation showed that besides *cis*-accumulation, there is extensive *trans*-accumulation in neuronal cells as well. The same was observed in PAS-A NPCs as well, albeit less strongly (Supplementary Figure S2A). To assess nuclear RNA levels across the entire 220-kb polycistron, we performed RNA-seq on purified nuclei. The RNA-seq confirmed that specific portions of Meg3 RNA are present at high levels in the nucleus. It also revealed that processed forms of Rian snoRNA were present at high concentration in the nucleus as well, in agreement with an earlier report on the *cis*-accumulation of snoRNAs in neural cells (50). Although in PAS-A1 and PAS-A2 NPCs there was still considerable presence of specific Meg3 and Rian sequences in the nucleus, overall RNA levels across the polycistron were reduced compared to WT NPCs, in accordance with the RT-qPCR analyses of total-RNA samples (Supplementary Figure S2B).

We next assessed *Dlk1*, which becomes activated primarily on the paternal allele during stem cell differentiation (15,22). *Dlk1* mRNA levels were about twice as high in the PAS-A1 and PAS-A2 NPCs compared to control NPCs (Figure 1F). Concordantly, whereas in the control NPCs *Dlk1* expression was mostly paternal, the PAS-A1 and PAS-A2 NPCs showed expression from both the parental chromosomes (Figure 1G). To confirm the biallelic *Dlk1* activation (‘loss of imprinting’), we performed chromatin immunoprecipitation (ChIP) against histone H3 lysine-4 tri-methylation (H3K4me3), which marks active promoters. Whereas, as expected, H3K4me3 was strictly paternal at *Dlk1* in the control NPCs, it was detected on both the parental alleles in the PAS-A1 and PAS-A2 NPCs (Figure 1H). These findings demonstrate that the reduced transcription across the *Meg3-Rian-Mirg* polycistron in NPCs, either directly or indirectly through its RNA products, gave strongly reduced repression of *Dlk1* on the maternal chromosome.

To assess if mono-allelic activation of *Dlk1* in mesodermal lineages (15) was similarly perturbed, we applied a published procedure to generate cardiomyocytes (CMCs) from mESCs [(37), see Materials & Methods]. In WT control cells, at day 12 of differentiation, a high proportion of cells had differentiated into CMCs, with expression of cardiac Troponin-T (Supplementary Figure S3A). Concordantly, the cardiac-lineage markers *Tbx5*, *Gata4*, *Mesp1* and *Nkx2.5* were strongly upregulated as well, and a comparable marker-gene activation occurred in the PAS-A1 and PAS-A2-derived CMCs (Supplementary Figure S3B). In the PAS-A1 and PAS-A2 cardiomyocytes, Meg3, Rian and Mirg RNA levels were reduced by about 20% only (Supplementary Figure S3C). A concordant, minor effect on *Dlk1* imprinting was observed, with partial activation of the normally-silent maternal *Dlk1* allele (Supplementary Figures S3D, E). These observations in CMCs complement our finding in NPCs that transcription across the polycistron is important to prevent *Dlk1* activation *in-cis* on the maternal chromosome.

The pAS insertion study indicates that the expression level of the *Meg3* polycistron is critical for the repression *in cis* of *Dlk1* during differentiation.

### Rian C/D-box snoRNAs are not required for *Dlk1* imprinted expression

Earlier studies in NPCs and newborn mice have showed that deletion and overexpression of the *Mirg* miRNAs does not influence the level or allelism of expression of *Dlk1* (51–54). However, allele-specific functional studies on the *Rian* snoRNAs have not been reported. Since processed forms of Rian are locally retained in the nucleus (50), and we still detected substantial expression in the PAS-A NPCs, our pAS insertion study did not exclude a possible involvement of *Rian* in *Dlk1* imprinting.

**Figure 2.**
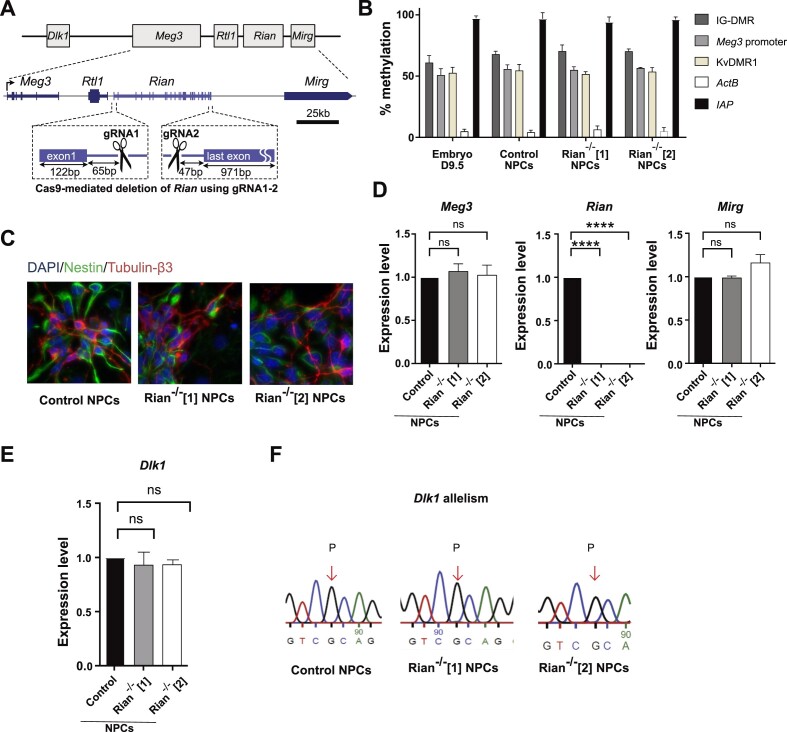
Deletion of the Rian snoRNA cluster does not affect *Dlk1* imprinting. (**A**) Schematic presentation of the CRISPR-Cas9-mediated *Rian* deletion in mESCs. (**B**) DNA methylation status in hybrid mESC-derived NPCs at d12 of neural differentiation (control and *Rian^−/−^*) and in E9.5 embryo as determined by methylation-sensitive qPCR. Bars represent means ± SD from three experiments. (**C**) IF staining of Nestin (green) and Tubulin-β3 (red) with DAPI counterstaining (blue) in NPCs at d12 of neural differentiation. (**D**) RNA accumulation of the *Meg3, Rian* and *Mirg* ncRNAs (RT-qPCR on total RNA samples) relative to housekeeping genes (*β-actin* and *Gapdh*) in control, Rian^−/−^[1] and Rian^−/−^[2] NPCs. Bars represent means ± SD from three independent experiments (ns, non-significant; **** *p*< 0.0001). (**E**) *Dlk1* mRNA amounts in control, Rian^−/−^[1] and Rian^−/−^[2] NPCs (ns, non-significant). (**F**) Sanger sequencing-based assessment of the allele-specificity of *Dlk1* expression in control, Rian^−/−^[1] and Rian^−/−^[2] NPCs. The arrow indicates the SNP used to distinguish maternal (M) and paternal (P) alleles.

To address this question, we deleted a region of 56 kb comprising the entire snoRNA locus in the hybrid mESCs, using a CRISPR-Cas9 NHEJ-mediated deletion approach (Figure 2A). Two independent mESC lines with biallelic *Rian* deletion—called Rian^−/−^[1] and Rian*^−/−^*[2]—were selected. Both these knock-out mutants had unaltered DNA methylation at the *Meg3* DMR and the IG-DMR (Supplementary Figure S4A). As expected, Rian RNA was no longer detected by RT-qPCR analysis in the Rian^−/−^[1] and Rian^−/−^[2] mESCs, whereas the amounts of the other ncRNAs of the polycistron remained at levels similar to control cells (Supplementary Figure S4B). RNA-seq on nuclear RNA confirmed that *Rian* was deleted in the Rian^−/−^[1] mESCs, with a complete lack of RNA signal across the entire *Rian* gene, and that the remainder of the polycistron remained expressed (Supplementary Figure S5C).

**Figure 3.**
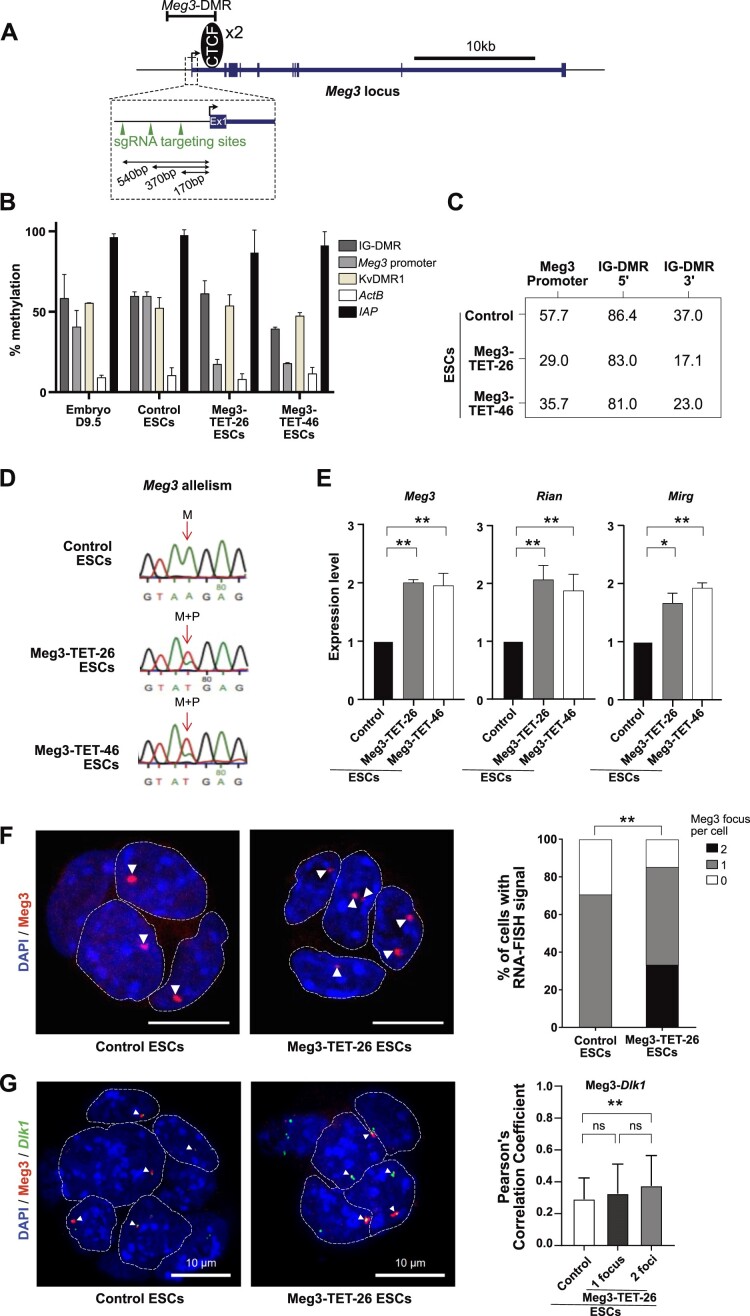
dCas9-suntag-TET1 mediated demethylation of the *Meg3*-DMR induces biallelic expression of Meg3 in mESCs. (**A**) Schematic presentation of *Meg3*, with the gRNAs used for promoter demethylation in mESCs. (**B**) DNA methylation status in hybrid mESCs (control, Meg3-TET-26 and Meg3-TET-46) and in E9.5 embryos, determined by methylation-sensitive qPCR. Bars represent means ± SD from three experiments. (**C**) DNA methylation status in hybrid mESCs (control, Meg3-TET-26 and Meg3-TET-46) as determined by pyrosequencing. Data represent the percentile methylation levels of 3 CpG dinucleotides at *Meg3* promoter, 5 CpG at the 5′ part of the IG-DMR and the 11 CpGs at the 3′ part of the IG-DMR. (**D**) Sanger sequencing-based assessment of the allelism of *Meg3* expression in control, Meg3-TET-26 and Meg3-TET-46 mESCs. The arrow indicates the SNP used to distinguish the maternal (M) and paternal (P) alleles. (**E**) RNA accumulation of the *Meg3*, *Rian* and *Mirg* ncRNAs (RT-qPCR) relative to housekeeping genes (*β-actin* and *Gapdh*) in control, Meg3-TET-26 and Meg3-TET-46 mESCs. Bars represent means ± SD from three experiments (** *P*< 0.01, * *P*< 0.05). (**F**) RNA-FISH analysis of the Meg3 lncRNA in control mESCs (*n* = 140) and Meg3-TET-26 mESCs (*n* = 123). DNA was counter-stained with DAPI (blue); nuclei are delineated by a dashed line; scale bar, 10 μm. On the right: the proportion of nuclei with zero, one or two Meg3 focus. (∗∗ *P* < 0.005, two-sided Fisher's exact test). (**G**) Combined RNA + DNA FISH detection of Meg3 RNA (red) and the *Dlk1* gene (green, with a fosmid probe) in control and Meg3-TET-26 ESCs. DNA was counter-stained with DAPI (bleu). Arrows show overlap/proximity between Meg3 and *Dlk1*. Dashed lines demarcate projection nuclei with foci; scale bar, 10 μm. To the right: Pearson coefficients for Meg3-*Dlk1* overlap calculated for control ESCs (*n* = 62, maternal chromosome), and for Meg3-TET-26 ESCs with a single RNA accumulation spot (*n* = 33; ‘1 focus’) or two RNA spots (*n* = 77; ‘2 foci’, both parental chromosomes).

Rian^−/−^[1] and Rian*^−/−^*[2] mESCs differentiated normally into NPCs, and showed unaltered DNA methylation at the IG-DMR and the *Meg3* promoter (Figure 2B), and a pattern of Nestin and Tubb3 expression similar to control NPCs (Figure 2C). RT-PCR analysis also confirmed normal expression of the neural marker genes *Nestin*, *Fabp7* and *Emx1* in the Rian-/-[1] and Rian^−/−^[2] NPCs as compared to control NPCs (Supplementary Figure S4D). The *Rian^−/−^* NPCs did not express *Rian*, as expected, and had unaltered levels of the Meg3 and Mirg RNAs (Figure 2D). *Dlk1* expression levels and the paternal allele-specificity of *Dlk1* expression were unaltered in the *Rian^−/−^* NPCs as well (Figure 3E, F). Together, these findings demonstrate that the Rian C/D-box snoRNAs do not control the imprinted expression of *Dlk1*.

Combined, our analyses of the pAS insertion and the *Rian^−/−^* mESC lines indicate that transcription across the polycistron is important for the acquisition of *Dlk1* imprinted expression, but that this function does not require the expression of *Rian*, located downstream of *Meg3*. Given that *Mirg* miRNAs also does not influence the level or allelism of expression of *Dlk1* (51–54), we conclude that the lncRNA Meg3 itself is required for *Dlk1* imprinted expression, with its expression levels influencing the degree of the in*-cis* repression.

### CRISPR-mediated demethylation of the *Meg3* promoter induces biallelic ncRNA expression

Temple Syndrome (TS14) is a human imprinting disorder (ID) characterized by increased expression of the ncRNA polycistron, affecting MEG3, MEG8 (called Rian in the mouse) and MIRG RNA levels (55–57). This aberrant ncRNA expression could have a causative involvement in this developmental and aberrant-growth disorder; yet, the underlying mechanisms remain largely unknown (57). TS14 often arises through maternal uniparental disomy of chromosome 14 (mUPD14)—where the human *DLK1-DIO3* locus resides—leading to biallelic *MEG3* expression (55). Less frequently, the syndrome is caused by losses of methylation at the *MEG3* promoter region (24,58–60). To model the increased biallelic *MEG3* expression in TS14 expression and to unravel its molecular effects, we performed CRISPR-dCas9-based epigenetic editing in our hybrid mESCs. Particularly, we asked whether demethylation of the (paternal) *Meg3* promoter (DMR) is sufficient to induce biallelic *Meg3* lncRNA expression, and, if so, whether this gives rise to biallelic repression of *Dlk1* during ESC differentiation.

We used a dCas9-SunTag system that recruits up to ten copies of the catalytic domain of TET1 to the targeted DNA sequence and neighboring DNA (38). Recently, this transient approach was applied to demethylate the intergenic ICR of the *Igf2-H19* imprinted domain in mESCs (38,61). To benchmark the technology, we therefore first aimed to demethylate the *H19* ICR in our hybrid mESC system. For this purpose, we designed a single guide-RNA directed against the second CTCF binding site in the *H19* ICR, which is methylated on the paternal chromosome only (Supplementary Figure S5A). To screen for maintained DNA hypomethylation in the obtained clones, we used a restriction-endonuclease digestion approach to quantify DNA methylation at CTCF binding sites 2 and 4, and at the *H19* promoter. Two of the clones—H19-TET-2.1 and H19-TET-2.7—showed strongly reduced methylation levels at all three regions analysed, with only about 20% of remaining methylation (Supplementary Figure S5B). For CTCF site-2 and the *H19* promoter, we confirmed hypomethylation by pyrosequencing and by reduced representation bisulfite sequencing (RRBS) (Supplementary Figure S5C,D). Following differentiation of H19-TET-2.1 and H19-TET-2.7 cells into CMCs, the *H19* ICR and the *H19* promoter faithfully retained their hypo-methylated state, with the same low methylation levels detected as in the mESCs from which they derived (Supplementary Figure S5E). In contrast, in H19-TET-2.1 and H19-TET-2.7 derived NPCs we noted a regain of normal methylation levels (Supplementary Figure S5E).

With experimental conditions in place, we next set out to demethylate the *Meg3* promoter. For this, we used three guide RNAs that target the *Meg3* promoter within an interval of 380 bp (Figure 3A). Two mESC lines that showed *Meg3* demethylation—Meg3-TET-26 and MEG3-TET-46—were selected for further studies (Figure 3B). As confirmed by pyrosequencing, these lines showed an about 50% reduction of methylation at the *Meg3* promoter (Figure 3C). We used RRBS as another approximate of methylation levels. This confirmed that the *Meg3* promoter/CpG island had become hypomethylated in both the Meg3-TET-26 and Meg3-TET-46 mESC lines, in a genomic interval comprising the *Meg3* promoter, exon 1 and part of intron-1 (Supplementary Figure S6A). At the IG-DMR, the 5′-side retained its high methylation level, whereas a partial reduction in methylation was observed at the 3′ side of the IG-DMR (Figure 3C). This sensitivity of the 3′ side of the IG-DMR to the targeted demethylation, despite the genomic distance from the *Meg3* promoter (∼13 kb), may be due to its reported physical proximity with the *Meg3* promoter within the nucleus (7).

**Figure 4.**
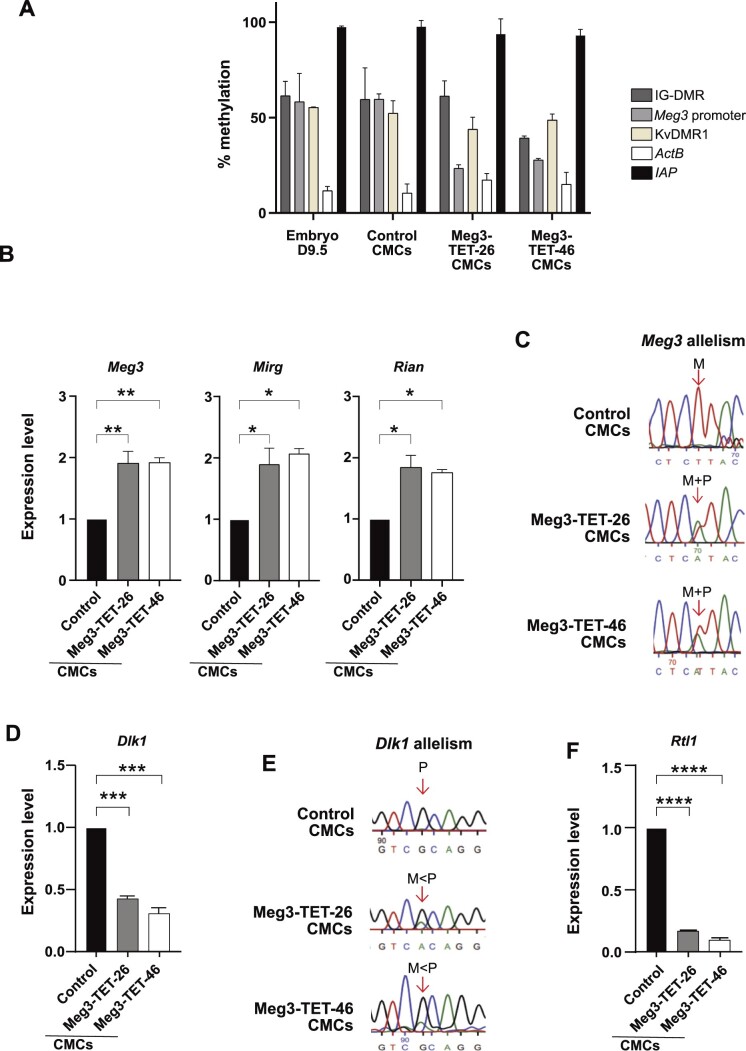
Demethylation of the *Meg3* DMR affects *Dlk1* and *Rtl1* imprinted expression. (**A**) DNA methylation status in hybrid mESC-derived CMCs (control, Meg3-TET-26 and Meg3-TET-46) and in E9.5 embryos as determined by methylation-sensitive qPCR. Bars represent means ± SD from three experiments. (**B**) RNA accumulation of the *Meg3*, *Rian*, and *Mirg* ncRNAs (RT-qPCR) relative to housekeeping genes (*β-actin* and *Gapdh*) in control, Meg3-TET-26 and Meg3-TET-46 CMCs. Bars represent means ± SD from three experiments (** *P*< 0.01, * *P*< 0.05). (**C**) Sanger sequencing-based assessment of *Meg3* expression allelism in control, Meg3-TET-26 and Meg3-TET-46 CMCs. (**D**) *Dlk1* mRNA amounts in control, Meg3-TET-26 and Meg3-TET-46 CMCs (*** *P*< 0.001). (**E**) Sanger sequencing-based assessment of *Dlk1* allelism in control, Meg3-TET-26 and Meg3-TET-46 CMCs. (**F**) *Rtl1* mRNA amounts in control, Meg3-TET-26 and Meg3-TET-46 CMCs (****P< 0.0001).

In agreement with the induced *Meg3* promoter hypo-methylation, the Meg3-TET-26 and Meg3-TET-46 mESCs showed biallelic *Meg3* expression (Figure 3D). Meg3, Rian and Mirg RNA levels were about 2-fold higher than in control ESCs (Figure 3E). To confirm the allelism of *Meg3* expression, we performed RNA FISH. In WT BJ ESCs, a single accumulation focus was apparent in about 70% of the cells—with no cells showing two foci—similarly as reported before (22). On the contrary, about 30% of the Meg3-TET-26 cells showed two nuclear accumulation foci, with a similar retention on the locus on both the parental chromosomes, in agreement with the TET1-induced partial demethylation of the paternal *Meg3* promoter (Figure 3F). Next, we performed RNA-FISH against Meg3 RNA combined with DNA FISH against *Dlk1*. In the Meg3-TET-26 ESCs that showed two Meg3 RNA foci (expression from both parental chromosomes), there was a similar overlap with *Dlk1* as in the WT cells with a single focus (maternal expression only) (Figure 3G). Although this assay does not tell the parental chromosomes apart, this observation suggests that in Meg3-TET cells with two RNA foci, the Meg3 *cis*-accumulation was comparable on the maternal and the paternal chromosomes.

### CRISPR-induced biallelic *Meg3* expression leads to biallelic *Dlk1* repression and loss of *Rtl1* expression in differentiated cells

Having generated ECSs with *Meg3* biallelic expression, we next explored its effects on the *Dlk1* expression in differentiated cells. The Meg3-TET-26 and Meg3-TET-46 mESCs could be readily differentiated into CMCs with stable maintenance of the *Meg3* hypomethylation (Figure 4A), similarly as observed for the CRISPR-demethylated *H19* ICR in CMCs (Supplementary Figure S5E). Consequently, *Meg3* expression remained biallelic in the differentiated Meg3-TET-26 and Meg3-TET-46 cells, and this correlated with a 2-fold increase in Meg3, Rian and Mirg RNA amounts (Figure 4B, C).

In the *Meg3*-hypomethylated CMCs, the biallelic *Meg3* expression and lncRNA *cis*-accumulation correlated with strongly reduced *Dlk1* expression levels (Figure 4D) with, as expected, the maternal allele being less strongly expressed than the paternal (Figure 4E). Combined, the above findings show that the induced loss of methylation at the *Meg3* DMR had induced biallelic *Meg3* expression, which gave *Dlk1* repression on both the parental chromosomes.

Meg3-TET-26 and Meg3-TET-46 derived NPCs were studied as well, and showed expression of the neural markers Nestin and Tubulin-β3 as in control NPCs (Supplementary Figure S6B). However, in these neural cells, similarly as was observed at the *H19* ICR in the H19-TET NPCs (Supplementary Figure S5E), we noted that the *Meg3* DMR was methylated at levels similar as in the control NPCs (Supplementary Figure S6C). Concordantly, *Meg3* expression levels were as in the control WT NPCs, with normal maternal allele-specific expression (Supplementary Figure S6D, E). Consequently, in the Meg3-TET-26 and Meg3-TET-46 derived NPCs, we noted unaltered levels of *Dlk*1 expression, from the paternal chromosome only, precisely as in the NPCs derived from the WT control mESCs (Supplementary Figure S6F, G). Given the aberrant regain of methylation at the *Meg3* DMR, the Meg3-TET-26 and Meg3-TET-46 derived NPCs were not used for further imprinting studies.

Next, we explored *Rtl1*, an imprinted gene that overlaps the *Meg3–Rian–Mirg* polycistron, but that is transcribed in the opposite direction (Figure 1A). The *Rtl1* gene is not expressed on the maternal chromosome, where the *Meg3* promoter drives the expression of the polycistron (16). Besides its main site of expression, the placenta, *Rtl1* is expressed in muscle cells where its deficiency causes distinct muscle abnormalities in mice (40).

Using a published strand-specific RT-PCR approach (62), Rtl1 mRNA was readily detected in the mESC-derived cardiomyocytes. In the Meg3-TET-26 and Meg3-TET-46 CMCs, in contrast, hardly any *Rtl1* RNA was detected (Figure 4F). Thus, the biallelic high transcription across the ncRNA polycistron—including across *Rtl1*—had given an almost complete loss of *Rtl1* expression. Though we could not distinguish the parental alleles, the loss of *Rtl1* expression implies that the CRISPR-induced *Meg3* promoter activity and polycistron transcription lead to a loss of *Rtl1* expression on the paternal chromosome, precisely as it does on the maternal chromosome (16).

### Differential *Meg3* expression guides parental chromosome-specific sub-TAD organization

The *Dlk1-Dio3* domain is organized into parent-of-origin specific 3D chromatin organizations, resulting in the formation of allele-specific sub-Topologically Associating Domains (sub-TADs) that contribute to the imprinted gene expression (30). Particularly, using an allelic 4C-seq approach, we recently reported that the maternal chromosome is organised into a CTCF-structured sub-TAD that coalesces the *Dlk1* and the *Meg3* promoters and that this contributes to the regulation of *Dlk1* imprinting (30). Whether the allelic *Meg3* expression and/or its methylation status instruct the functionally relevant differential sub-TAD organization is unknown.

**Figure 5.**
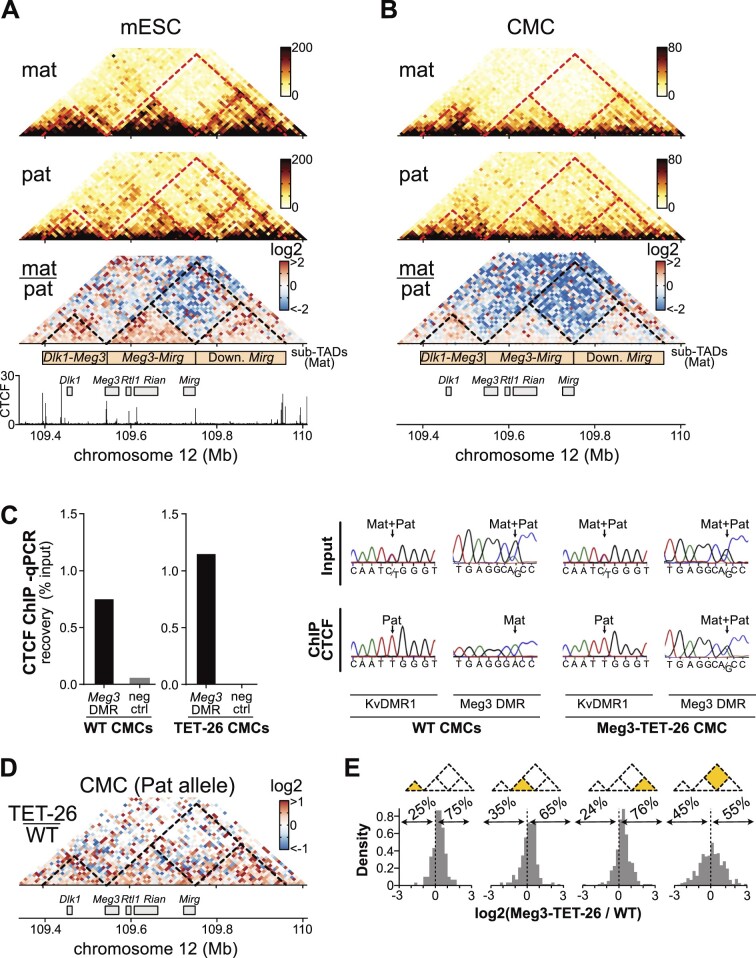
*Meg3* DMR demethylation causes reorganization of sub-TADs within the *Dlk1-Dio3* domain. (**A**) 3D chromatin organization in hybrid mESCs determined by allelic Capture-C for the maternal (top) and the paternal chromosome (middle). A comparison matrix is shown below (log_2_ ratio). Bins are 10-kb. The matrixes are aligned with CTCF ChIP-seq in WT control mESCs. The interrupted lines outline maternal-specific sub-TADs, similarly as described before (30). (**B**) 3D chromatin organization in hybrid mESC-derived CMCs, as determined by Capture Hi-C. From top to bottom: the maternal allele, the paternal allele and the comparison matrix (log_2_ ratio). (**C**) Left: CTCF ChIP on control and Meg3-TET-26 CMCs. Percentile precipitation was determined by qPCR at ‘binding site 2′ in the *Meg3* DMR. Right: Sanger sequencing profiles assess the allele-specificity of CTCF binding at the *Meg3* DMR and the KvDMR1. (**D**) Comparison of the 3D chromatin organization between the paternal allele of hybrid mESC-derived control and Meg3-TET-26 CMCs (Capture Hi-C). In the comparison matrix (log_2_ ratio), stronger signal in the TET-26 CMC is shown in red, while stronger signal in control is shown in blue. Dashed black lines outline the maternal-specific sub-TADs. (**E**) Distribution of log_2_ ratios between control and Meg3-TET26 CMCs for all bins comprised in the four zones highlighted in yellow. The percentage of bins with positive/negative log2 ratio is indicated. For the three zones along the x-axis, the histogram values are skewed towards positive values, indicative of increased short-range interactions in Meg3-TET-26 CMCs. On the contrary, the histogram is centered close to zero in the rightmost panel, representing the more distal interactions between the *Meg3–Rian* sub-TAD and the downstream *Mirg* sub-TADs.

To resolve better the sub-TAD organization along the entire imprinted domain, we used allelic Capture-C and Capture Hi-C approaches (Figure 5). The overarching region of interest was ‘captured’ using probes that covered a 650-kb region comprising *Dlk1* and the entire *Meg3–Rian–Mirg* polycistron (see Materials and methods). Similarly as in our recent 4C-seq study (30), in the hybrid WT mESCs we detected a strong sub-TAD on the maternal chromosome that comprised both the *Dlk1* gene and the *Meg3* promoter (‘*Dlk1-Meg3* sub-TAD’) (Figure 5A). The large majority of the polycistron itself is contained within another sub-TAD, which is most prominent on the maternal chromosome as well, and appears delineated by CTCF binding sites in *Meg3* promoter region [maternal-specific; (30)] and extends to downstream of the *Mirg* mRNA cluster (Figure 5A). Importantly, we find that the differential sub-TAD organization between the parental chromosomes persists during ESC differentiation into CMCs (Figure 5B). Concordantly, maternal-specific binding of CTCF was observed at *Meg3* promoter (intron-1) in the WT CMCs, as in WT mESCs (30) (Figure 5C).

CTCF binding does not occur when critical CpG dinucleotides within its binding motif are methylated (63,64). We therefore verified if biallelic CTCF binding occurred after inducing hypomethylation at the paternal *Meg3* DMR. Indeed, a more pronounced CTCF precipitation was observed in the Meg3-TET-26 CMCs, with CTCF binding now detected at both the maternal and the paternal *Meg3* DMR (Figure 5C). This finding shows that the CRISPR induced hypomethylation, which persisted upon differentiation (Figure 4A), led to biallelic CTCF binding to the *Meg3* promoter region in the CMCs.

Next, we assessed the impact of the induced loss of methylation, the gain of CTCF binding and the gain of *Meg3* expression on the 3D-organization of the paternal chromosome. For this, we compared WT and *Meg3*-hypomethylated cells. Initial 4C-seq observations in mESCs, using three different viewpoints, showed that the paternal allele of hypomethylated cells exhibited stronger interactions within the *Dlk1-Meg3* sub-TAD (Supplementary Figure S7A, yellow highlighted area). Quantification confirmed this visual observation, with the 3D-interaction patterns of the hypomethylated paternal allele being intermediate between those of the maternal allele and those of the WT paternal allele (Supplementary Figure S7B, violet bars *versus* blue/red bars). To corroborate this finding in CMCs, we used Capture Hi-C to compare the 3D-organization between WT and the Meg3-TET-26 CMCs. This revealed altered interaction frequencies on the paternal chromosome upon loss of *Meg3* methylation. Notably, there was an increase in short-range interactions (1.5-fold) in the Meg3-TET-26 CMCs (Figure 5D, positive log2 ratio illustrated by red pixels). This overall trend was observed across the entire domain. This was notably the case in the two sub-TADs that hinge the *Meg3* promoter, but also in a sub-TAD downstream of *Mirg*. Quantifications confirmed this pattern, with the three sub-TADs displaying stronger interaction in Meg3-TET-26 CMCs compared to WT CMCs (Figure 5E, log_2_ score skewed for positive values). These data show that the paternal chromosome of the Meg3-TET-26 CMCs adopted a more maternal-like 3D-organization. This finding agrees with the observed increase in repression of *Dlk1* on the paternal chromosome, more resembling the maternal chromosome as well (Figure 4D, E).

We conclude that the allelic methylation status of the *Meg3-*DMR dictates the allelic binding of CTCF, and thereby instructs differential sub-TAD organization, and this correlates with *Dlk1* imprinted gene expression.

Similar to CMCs, the organization of the maternal *Dlk1-Dio3* domain into sub-TADs was maintained in normal mESC-derived NPCs as well (Supplementary Figure S7C), albeit with a weaker insulation between maternal sub-TADs (Supplementary Figure S7C versus Figure 5B). The premature termination of *Meg3* transcription by the insertion of a poly-adenylation site in intron 1 (Figure 1, PAS-A1 cells) did not perturb CTCF binding at the *Meg3* DMR, which remained exclusively maternal (Supplementary Figure S7D).

A moderate re-organization of the 3D-organization was observed by Capture Hi-C in the PAS-A1 NPCs on the maternal chromosome. In these neural cells with strongly reduced Meg3 lncRNA expression (Figure 1E), increased short-range interactions were observed within the *Dlk1-Meg3* sub-TAD (Supplementary Figure S7E, red pixels illustrative of positive log2 ratio, confirmed by quantifications) while no clear trends were observed in the two other sub-TADs of the domain (Supplementary Figure S7F, equal mix of positive and negative log_2_ ratios). The strongly reduced Meg3 RNA levels in PAS-A1 NPCs (Figure 1E) was thus associated with increased short-range interactions only in the sub-TAD upstream of *Meg3* TSS. The abundance of Meg3 transcript therefore seemed less critical than that of the *Meg3* DMR methylation level for the establishment and maintenance of the parental-specific sub-TADs covering the imprinted domain.

Our study does not address the function of Meg3 on its own, and how this lncRNA could repress the *Dlk1* gene in *cis*. Previously, we reported that the PRC2 component EZH2 -which mediates H3K27me3- is essential for the imprinted expression of *Dlk1* (22). Meg3 lncRNA seems to interact with the PRC2-associated proteins EZH2 and JARID2 (65,66), similarly as was reported for many other long nascent RNAs (67). These observations suggested that Meg3 could control *Dlk1* by influencing PRC2-mediated H3K27me3. To explore this possibility, we performed ‘CUT&RUN’ experiments to assess H3K27me3 levels at *Dlk1* in normal and PAS-A1 NPCs (Supplementary Figure S8). H3K27me3 enrichment was detected at the *Hoxa11* locus as expected (positive control), compared to IgG and a negative control region (*Actin-B*) (*P*= 0.0014 and *P*= 0.0003 by Kruskal–Wallis test, in control and PAS-A1 NPCs respectively) (Supplementary Figure S8A, C). At the *Dlk1* promoter, H3K27me3 enrichment was detected in control and PAS-A1 NPCs, with signal on both the parental chromosomes. However, despite a tendency for higher H3K27me3 signal for the *Dlk1* promoter region in PAS-A1, no significant difference was observed. These observations extend an earlier ChIP-based study on ESCs and NPCs, in which depletion of Meg3 did not alter EZH2 levels at the *Dlk1* promoter and did not give rise to loss of H3K27me3 (22).

In the Meg3-TET-26 CMCs -in which the Meg3 RNA was expressed and retained on both the parental chromosomes- there was no apparent change in biallelic low H3K27me3 levels at the *Dlk1* promoter compared to control CMCs, despite significant enrichment at *Hoxa11* (*P*= 0.0022 and *P*= 0.0013, by Kruskal–Wallis test) in control and Meg3-TET-26 CMCs respectively (Supplementary Figure S8B, C).

It remains to be determined whether Polycomb group proteins are important to retain the Meg3 lncRNA on the maternal chromosome -possibly in conjunction with the observed 3D chromatin structural interactions- and whether Meg3 lncRNA acts on regulatory protein complexes at the *Dlk1-Dio3* domain, including the PRC1 complex.

## Discussion

**Figure 6.**
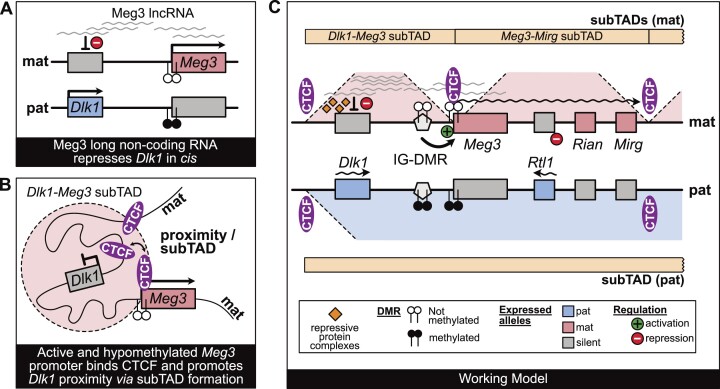
Model of how Meg3 lncRNA expression controls imprinted gene expression. Our study provides two main insights: (**A**) Meg3 lncRNA expression—and not *Rian*—controls the maternal allele-specific repression of the *Dlk1* gene during differentiation, and (**B**), Hypomethylation of the *Meg3*-DMR (on the active maternal *Meg3* allele) enhances the binding of CTCF to this DMR, thereby inducing a sub-TAD that brings *Dlk1* in close proximity to *Meg3* (and its lncRNA). (**C**) A model combining this study's and earlier insights: on the maternal chromosome, the ICR (called IG-DMR) is unmethylated and is an enhancer that activates the close-by *Meg3-Rian-Mirg* polycistron. Meg3 lncRNA and its partial retention on the locus control the allelic repression of *Dlk1*, and this may involve functionally important interactions with components of Polycomb Repressive Complexes (PRCs) (22,54,65,66) and/or other chromatin regulatory proteins (orange diamonds). Polycistron transcription downstream of *Meg3* prevents transcription of the developmental *Rtl1* gene, possibly through transcriptional interference. On the maternal chromosome, *Meg3* promoter activity protects against *de novo* acquisition of DNA methylation. It remains unmethylated and CTCF can bind to multiple recognition motifs at this DMR. As shown recently (30), the latter mediates about a sub-TAD structure that brings *Dlk1* in close proximity to *Meg3* (and Meg3 lncRNA) and these structural interactions contribute to the lncRNA-mediated *Dlk1* repression. On the paternal chromosome, the domain's ICR is fully methylated and lacks enhancer activity. The polycistron is thus not activated, leading to acquisition of *Meg3 de novo* methylation in the early embryo (80). The *Meg3*-DMR methylation, in turn, prevents CTCF binding and sub-TAD structuration. The lack of Meg3 lncRNA and absence of polycistron transcription allow the developmentally controlled *Dlk1* and *Rtl1* activation on this parental chromosome. In panel C, only CTCF binding sites at confirmed boundaries are depicted, including only one of the two CTCF sites upstream of *Dlk1* shown in panel B.

The main finding of this study is that *Dlk1* imprinting requires the lncRNA Meg3, and not the Rian snoRNAs, nor the act of transcription by itself alone. In the NPCs that had a poly(A) signal inserted into intron-1 of *Meg3*, the remaining lncRNA expression was insufficient to repress *Dlk1* on the maternal chromosome. In cardiomyocytes, a lesser reduction in lncRNA was achieved, and this had a concordant, smaller, effect on *Dlk1* imprinting. Our studies thus indicate that a certain level of lncRNA is required to prevent the transcriptional activation of *Dlk1* in-*cis* during differentiation. This provides a mechanistic model (Figure 6) as to why epigenetic and genetic changes affecting the *Meg3–Rian–Mirg* polycistron correlated with aberrant *Dlk1* expression in earlier mouse studies (6–8,10,20,22), and has implications for understanding human imprinting disorders that are linked to aberrant *MEG3* expression.

In a recent study on two other nuclear imprinted lncRNAs—Kcnq1ot1 and Airn—the level of lncRNA expression correlated with the degree of *in-cis* repression, and such a dosage effect was suggested to be linked to the formation of lncRNA-protein aggregates, possible involving liquid-liquid phase separation (68). Meg3, Airn and Kcnq1ot1 interact with components of Polycomb Repressive Complexes (PRCs), and these could thus be part of such aggregates (22,66,68–70). Notably, the PRC1 complex, via its subunit CBX2, can undergo phase separation and contributes to the formation of condensates (71,72). Further studies are required to determine if such aggregates form at the *Dlk1-Dio3* locus and to dissect the relative contribution of PRC1 and PRC2 and Meg3 lncRNA in this process.

Another key finding is that the *Meg3*-DMR methylation level instructs the structural organization of the *Dlk1-Dio3* domain, and mediates the formation of maternal chromosome-specific sub-TADs (Figure 6). We explored this aspect using a parental allele-specific Capture Hi-C approach. The activity of the *Meg3*-promoter keeps the CTCF binding sites of the *Meg3*-DMR unmethylated on the maternal chromosome, leading to CTCF binding on the maternal allele only (30,73). We, and others, demonstrated that this creates a maternal boundary that hinges the *Dlk1-Meg3* sub-TAD and a neighbouring sub-TAD comprising the ncRNA polycistron (7,30). We find here that the distinct sub-TAD organisation on the maternal chromosome is maintained during differentiation into CMCs or NPCs.

Importantly, in the Meg3-TET cardiomyocytes, in which the *Meg3* DMR was hypomethylated and active now on both the parental chromosomes, there was biallelic CTCF binding to the *Meg3* DMR (intron-1 region). Concordantly, in these differentiated cells the paternal chromosome acquired a sub-TAD structuration that was similar to that on the maternal chromosome, and this correlated with biallelic repression of *Dlk1* and *Rtl1*. Our results thus demonstrated that the methylation level of the *Meg3*-DMR is both instructive for CTCF binding, *Meg3* expression, sub-TADs structuration and the repression of paternally-expressed genes (Figure 6).

Notwithstanding the obtained insights, the Meg3-TET mESCs were sub-optimal since they still had some residual methylation at the *Meg3-*DMR. To set up the CRISPR-dCas9-SunTag-TET technology, we had initially demethylated the *H19* ICR in our hybrid ESCs. Similarly as in other studies (38,61), we achieved significant demethylation at the H19 ICR, but also detected about 10% residual methylation (Supplementary Figure S5). Our data suggest the possibility that at the *H19* ICR and the *Meg3* DMR the achieved demethylated state was not fully stable, leading to a WT methylation pattern in a small fraction of the cells. In addition, following differentiation of the Meg3-TET ESCs into NPCs, we noted that the *Meg3* DMR methylation level had become normal again, which prevented us from doing further studies on neural cells. In our system we could not determine whether the latter artefact was caused by re-acquisition of *de novo* methylation (61), or by positive selection of cells with normal *Meg3* methylation during the differentiation process. *Meg3* hypomethylation was stable upon differentiation into CMCs, however, suggesting that there had not been an advantage of a given methylation state in this embryonic lineage. More generally, our studies using the CRISPR-dCas9-SunTag-TET technology highlight the importance to use a culture medium that prevents acquisition of *de novo* DNA methylation in ESCs (34), and to ascertain that the achieved hypomethylation is stable in cell types of interest.

Combined, our different *Meg3* mutants clarified some of the functional interplays between *Meg3* expression, the 3D-organization of the locus and *Dlk1* regulation. In differentiated neural PAS-A1 cells, which had reduced levels of the lncRNA due to premature transcription termination within the first intron, we noticed unaltered allelic CTCF binding at the maternal *Meg3*-DMR. Concordantly, the sub-TADs remained largely similar, with even increased occurrence of short-range structural interactions within the sub-TAD upstream of *Meg3*-DMR on the maternal chromosome (Supplementary Figure S7). This suggests that the Meg3 lncRNA—which is partly retained in *cis* and spatially co-localizes with the *Dlk1* locus (22)—is largely dispensable for shaping the *Dlk1-Meg3* sub-TAD. Yet, we observed defective *Dlk1* repression in differentiated cells (Figure 1F), which may be linked to interactions between Meg3 RNA and components of PRC complexes, possibly through lncRNA-protein aggregates (3,54,65,66). We conclude that the accumulation of the Meg3 lncRNA is thus dispensable for CTCF binding at the *Meg3*-DMR and the maintenance of the maternal-specific sub-TADs. Further studies would be required to formally assessed if the RNA binding domains of CTCF—which appear important for some aspects of the higher order folding of chromatin (74,75) — are contributing to the maternal-specific sub-TADs insulation at the *Dlk1-Dio3* domains. Yet, our results already show that Meg3 lncRNA interplay with CTCF differs from HOTTIP lncRNA, which enhances CTCF recruitment at a subset of TAD boundaries thereby increasing their insulation (76), and also differ from Jpx lncRNA whose recruitment to chromatin antagonises CTCF recruitment to specific binding sites (77).

Our data on the Meg3-TET cells provide also additional insights on the imprinted *Rtl1* gene—an important regulator of placental and muscle development (40,78). In these cells, the CRISPR-induced expression of the *Meg3* polycistron on the paternal chromosome lead to a complete loss of *Rtl1* expression. This strongly suggests that—similarly as on the maternal chromosome—transcription of the polycistron across the *Rtl1* no longer allowed this gene to be expressed. Besides such a transcriptional interference effect, the reduction of Rtl1 mRNA levels may also result from increased levels of miRNAs, produced downstream of *Meg3*, that target the Rtl1 mRNA (16,40,78).

More generally, our study highlights the antagonism between the parental genomes at the *Dlk1-Dio3* imprinted domain. A sperm-derived paternal DNA methylation imprint at the intergenic IG-DMR keeps this ICR inactive on the paternal chromosome, thus preventing activation of the close-by *Meg3–Rian–Mirg* polycistron on this parental chromosome (7,79). The non-activated paternal *Meg3* promoter, consequently, becomes *de novo* methylated in the early embryo (7,10,80). On the maternal chromosome, in contrast, the intergenic ICR is unmethylated and transcriptionally active, producing enhancer RNAs (eRNAs) (8). The enhancer activates the maternal *Meg3* promoter, leading to the expression of Meg3 lncRNA, Rian snoRNAs and the Mirg and other miRNAs produced by the polycistron. The conserved lncRNA Meg3 (81) itself is important for the repression of *Dlk1* on the maternal chromosome, as shown in this study. Consequently, the transcriptional activation of *Dlk1* during cell differentiation occurs on the paternal chromosome only. As an additional layer of antagonism, several of the miRNAs produced by the maternally expressed *Meg3-Rian-Mirg* polycistron reduce the protein levels of the paternally expressed genes (82). For instance, one miRNA of the *Mirg* locus (miR-329) targets the 3′ UTR of *Dlk1* mRNA and inhibits its translation, thereby reducing DLK1 protein levels in neural cells (83).

In conclusion, different mechanisms evolved through which maternally-expressed non-coding RNAs antagonise paternally-expressed protein-coding genes, to limit their effects on growth, development and metabolism. Possibly affecting the same biological processes, several miRNAs of the domain antagonise paternally-expressed imprinted genes elsewhere in the genome (82). In neural cells, for instance, several of the *Mirg* cluster miRNAs reduce post-transcriptionally the expression of developmental paternally-expressed genes, including *Igf2* at the *Igf2-H19* domain, and the imprinted *Plagl1* transcription factor gene (52,82).

Our data may help to understand the molecular etiology of human imprinting disorders that are associated with altered *MEG3* expression. Particularly, we predict that in patients with Temple Syndrome (TS14) (29), and in some patients with the clinically overlapping Silver-Russell Syndrome (SRS) (24,84), their aberrant, biallelic *MEG3* expression would lead to strongly reduced DLK1 in tissues in which this non-canonical Notch ligand is imprinted. In addition, we predict loss of *RTL1* expression in muscle and other tissues where this retrotransposon-derived gene is expressed. In Kagami-Ogata Syndrome (KOS14), conversely, the loss of *MEG3* expression would correlate with increased *DLK1* and *RTL1* expression, now from both the parental chromosomes. In mice, DLK1 deficiency leads to fetal growth restriction and to metabolic and endocrinal defects (85–87). RTL1 deficiency in mice affects placental development and function (88) and leads to distinct muscle abnormalities. DLK1 and RTL1 are therefore likely main contributors to TS14 and KOS14, and recent clinical studies have started to explore this question (57,89).

## Supplementary Material

gkae247_Supplemental_File

## Data Availability

All sequencing data (4C-seq, Capture-C, Capture Hi-C, RNA-seq and RRBS) are available from the European Nucleotide Archive (EMBL-EBI ENA) repository under accession number PRJEB57653. The CTCF ChIP data used in this study are available in the NCBI GEO database, under accession number GSE207166.

## Notes

Present address: Sabina Farhadova, Department of Neuroscience, University of Wisconsin-Madison, Wisconsin, WI 53705, USA.

Present address: Amani Ghousein, Centre de Biologie Structurale and MIVEGEC, University of Montpellier, 34090 Montpellier, France.

Present address: Melisa Gomez-Velazques, Helmholtz Zentrum Muenchen, 85764 Neuherberg, Germany.
