# Supplementary material for: The long non-coding RNA Meg3 mediates imprinted gene expression during stem cell differentiation
Source: Nucleic Acids Res. 2024 Apr 13;52(11):6183–200. doi: 10.1093/nar/gkae247 (PMC11194098; doi:10.1093/nar/gkae247)
Supplement: gkae247_Supplemental_File [file gkae247_supplemental_file.pdf]

# The long non-coding RNA Meg3 mediates imprinted gene expression during stem cell differentiation

**Sabina Farhadova, Amani Ghousein, François Charon, Caroline Surcis, Melisa Gomez-Velazques, Clara Roidor, Flavio Di Michele, Maud Borensztein, Albertina De Sario, Cyril Esnault, Daan Noordermeer, Benoit Moindrot and Robert Feil.**

## SUPPLEMENTARY DATA:

*** Supplementary Figures S1-S8**

*** Supplementary Tables S1-S6**

***Supplementary References (cited in Supplementary Figures and Supplementary Tables)**

# SUPPLEMENTARY FIGURES

## -------------------------------------- Supplementary Figure S1 -----------------------------------

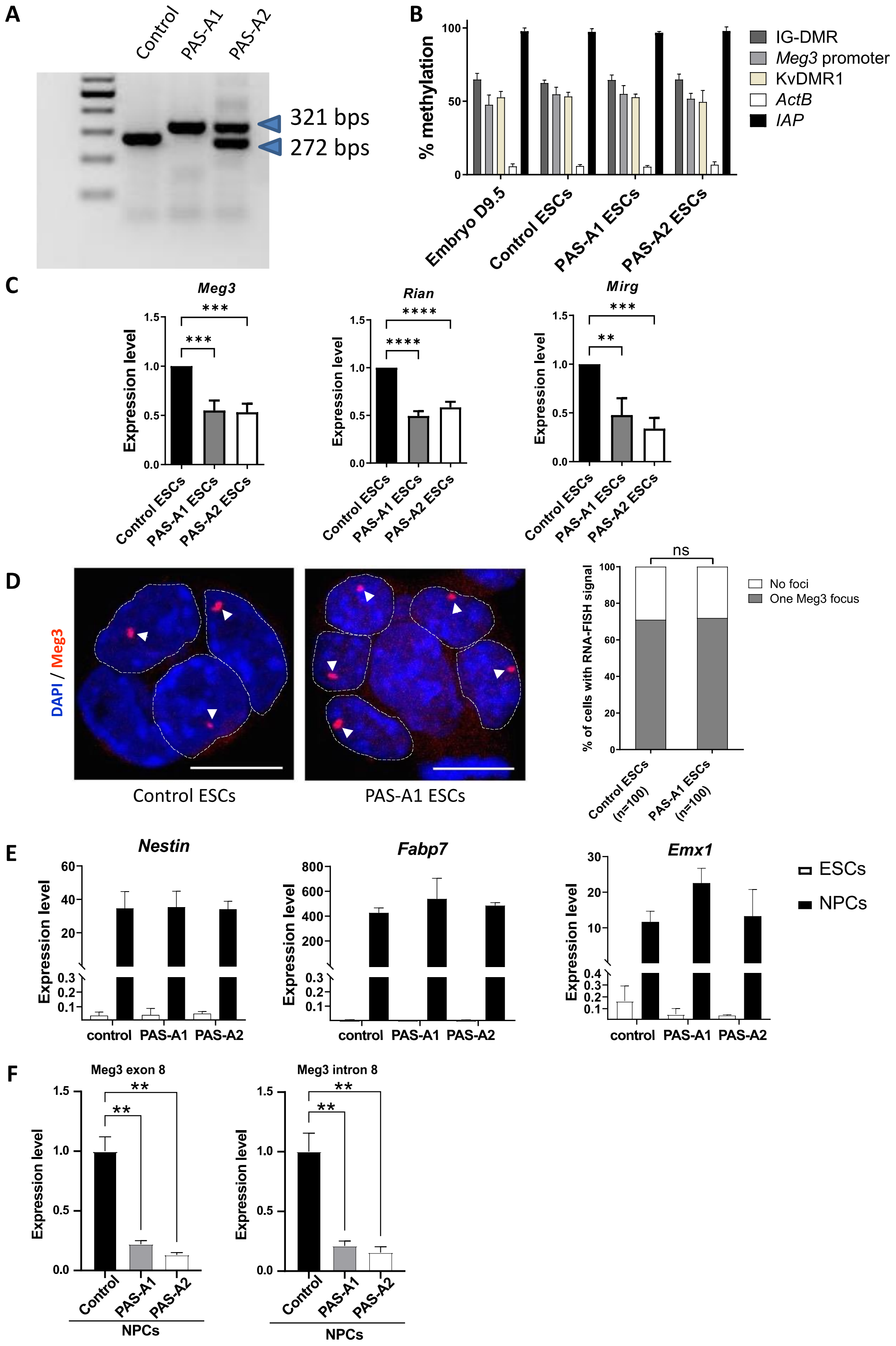

**Supplementary Figure S1. Meg3 intron-1 pAS insertion: DNA methylation and gene expression analysis.**

**(A)** Agarose gel showing PCR products from the region with the pAS insertion. Control mESCs show amplification of the WT allele (272-bp); PAS-A1 mESCs show biallelic pAS insertion (321-bp). PAS-A2 mESCs show mono-allelic pAS insertion (272-bp and 321-bp bands); on the maternal chromosome, assessed by Sanger-sequencing of the excised 321-bp band.

**(B)** DNA methylation status in hybrid mESCs (control, PAS-A1 and PAS-A2) and in E9.5 embryos, as determined by methylation-sensitive qPCR. Bars represent means ± SD from three experiments.

**(C)** RNA accumulation of Meg3, Rian and Mirg spliced-RNA levels assessed by RT-qPCR on total RNAs, relative to housekeeping genes (*β-actin* and *Gapdh*) in control, PAS-A1 and PAS-A2 NPCs. Bars represent means ± SD from three experiments (**** $p<0.0001$).

**(D)** RNA-FISH analysis of the Meg3 lncRNA in control mESCs (n=140) and PAS-A1 mESCs (n=222). DNA counter-staining with DAPI (blue); nuclei are delineated by a dashed line; scale bar, 10 $\mu$m. On the right: the proportion of nuclei without foci and one Meg3 focus. Data were analyzed by a two-sided Fisher's exact test (ns, non-significant).

**(E)** mRNA amounts of the neural marker genes *Nestin*, *Fabp7* and *Emx1* (RT-qPCR) in control, PAS-A1 and PAS-A2 mESCs and their derived NPCs, relative to two housekeeping genes (*β-actin* and *Gapdh*). Error bars represent the ± SD of three biological replicates.

**(F)** Meg3 RNA levels at exon-8 and at intron-8 relative to *β-actin* in control, PAS-A1 and PAS-A2 NPCs, assessed by RT-qPCR on total RNAs. Bars represent means ± SD from two biological replicates (** $p<0.01$).

---------------------------------------- **Supplementary Figure S2** ----------------------------------------

**A**

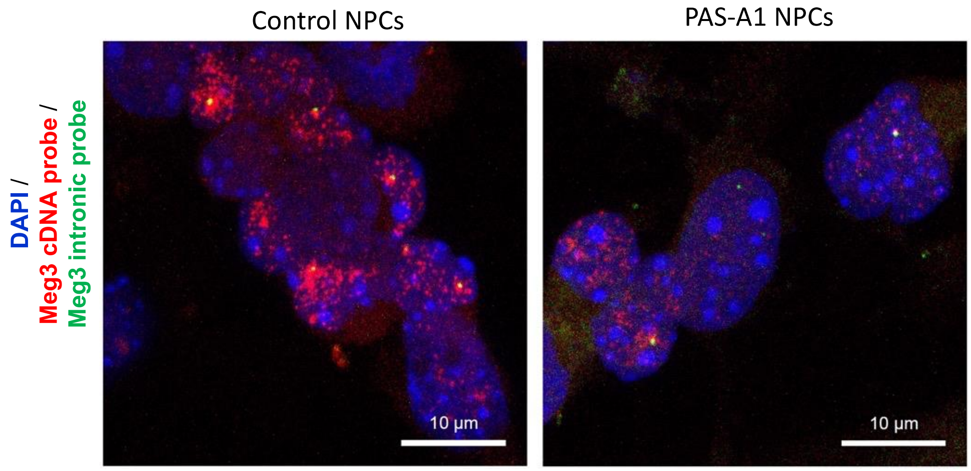


**B**

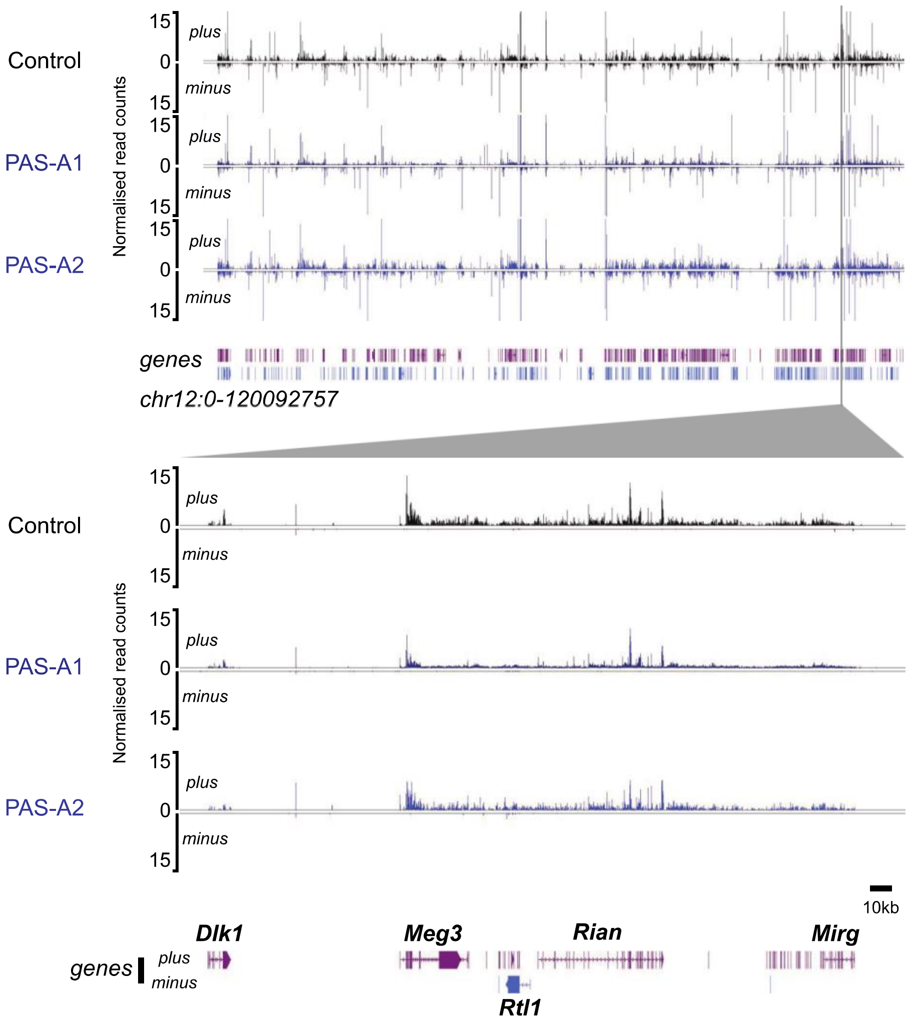

**Supplementary Figure S2. *Meg3* intron-1 pAS insertion: nuclear-RNA analysis.**

**(A)** RNA-FISH detection of Meg3 lncRNA in control NPCs, with a cDNA probe (in red) and with an intronic probe comprising intron 1 and 8 sequences (in green). Blue, DAPI counterstaining of DNA. Besides the RNA at the transcription site -the only signal detected with the intronic probe- most of the lncRNA accumulates within foci elsewhere in the nucleoplasm. Projection of 20 slices; scale bars, 10 µm.

**(B)** Nuclear RNA-seq on WT and PAS-A NPCs. Upper panel, chromosome 12 profiles corrected for spiked RNA (see Materials and Methods) for WT control, PAS-A1 and PAS-A2 NPCs. Lower panel, enlargement of the Meg3-Rian-Mirg polycistron, showing reduced expression in PAS-A1 and PAS-A2 relative to control NPCs. Based on corrected read counts, the estimated reduction in RNA along the polycistron is about 50% in PAS-A1 and 40% in PAS-A2.

---------------------------------------- **Supplementary Figure S3** ----------------------------------------

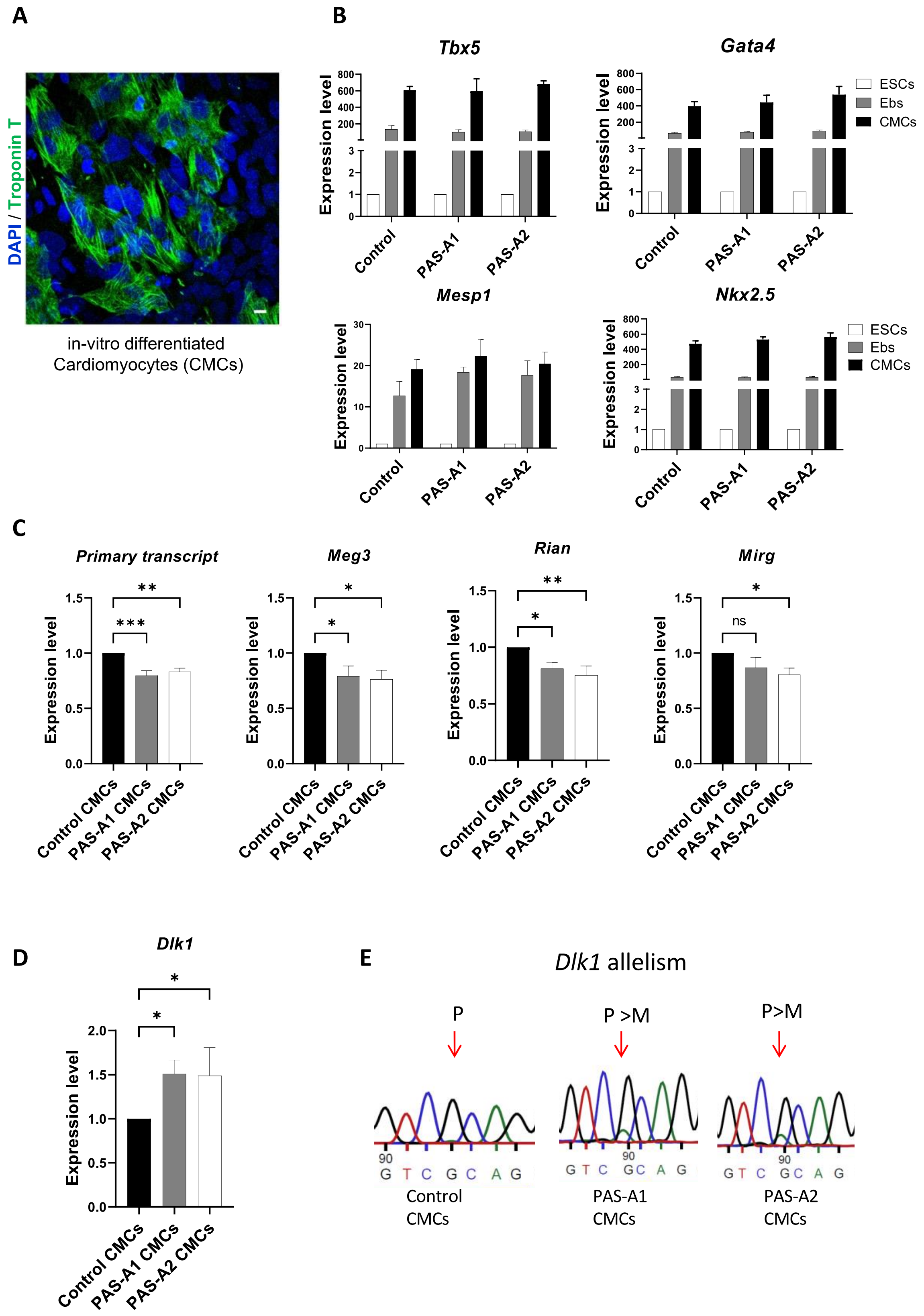

**Supplementary Figure S3. Meg3 intron-1 pAS insertion: effects on *Dlk1* imprinting in CMCs.**

**(A)** mESC-derived cardiomyocytes (CMCs) obtained following embryoid body (EB) formation and subsequent differentiation for 12 d, with IF staining of cardiac Troponin-T (green) and DAPI counter-staining (blue). Scale bar, 10 μm.

**(B)** mRNA amounts of the cardiac-lineage markers *Tbx5*, *Gata4*, *Mesp1* and *Nkx2.5* (RT-qPCR) in control, PAS-A1 and PAS-A2 mESCs, EBs and CMCs. Error bars represent the ± SD of three biological replicates.

**(C)** RNA accumulation of the *Meg3* primary transcript and the *Meg3*, *Rian* and *Mirg* ncRNAs (RT-qPCR), relative to two housekeeping genes (*β-actin* and *Gapdh*) in control, PAS-A1 and PAS-A2 CMCs. Bars represent means ± SD from three independent experiments (*** $p<0.001$, ** $p<0.01$, * $p<0.05$, ns = non-significant).

**(D)** *Dlk1* mRNA amounts in control, PAS-A1 and PAS-A2 CMCs. Bars represent means ± SD from three independent experiments (* $p<0.05$).

**(E)** Sanger sequencing-based assessment of the *Dlk1* expression allelism in control, PAS-A1 and PAS-A2 CMCs. The arrow indicates the SNP used to distinguish the maternal (M) and paternal (P) alleles.

## ------------------------------------ Supplementary Figure S4 ------------------------------------

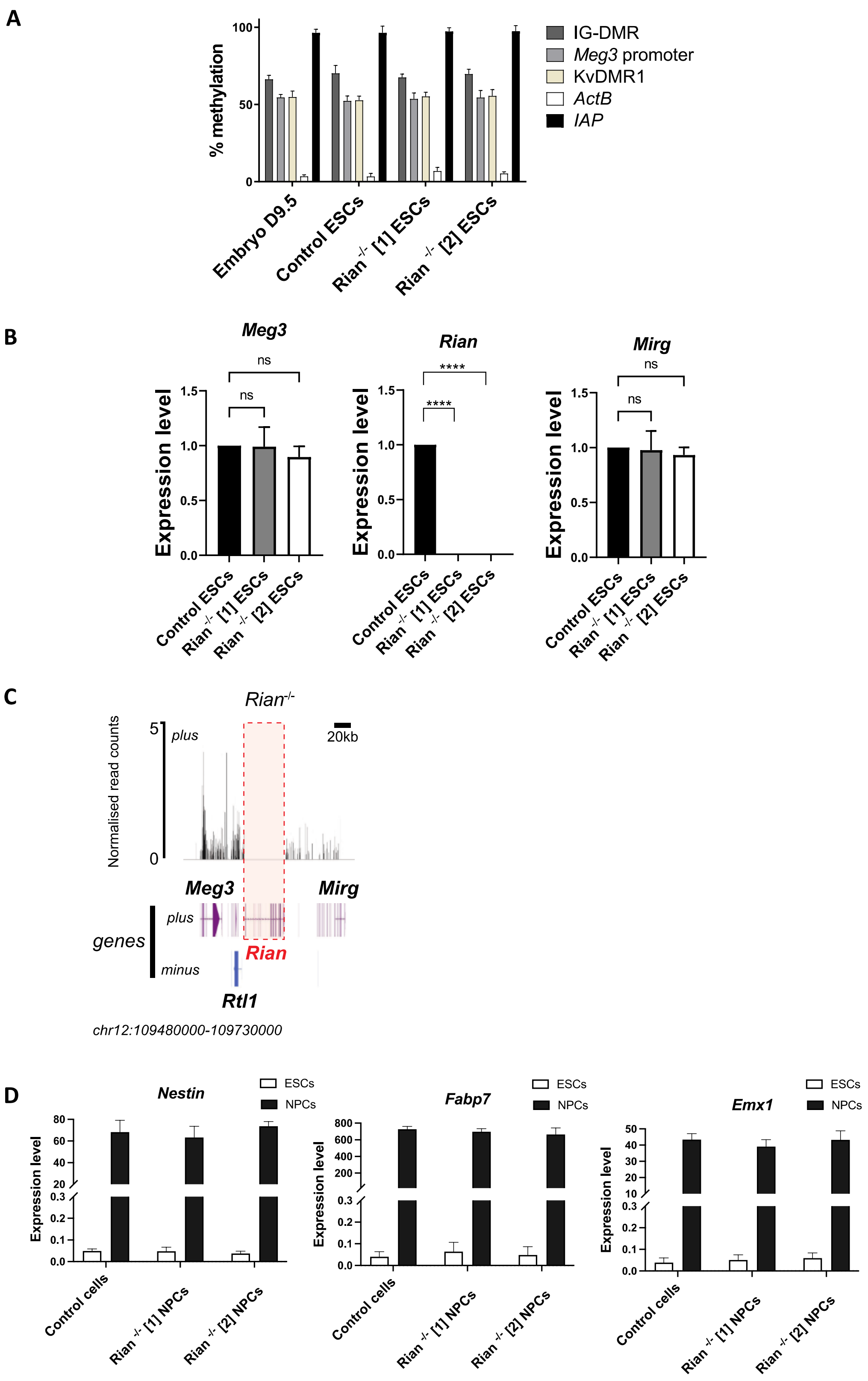

**Supplementary Figure S4. DMR methylation and gene expression in *Rian*$^{-/-}$ ESCs and NPCs.**

**(A)** DNA methylation status in hybrid mESCs (control, *Rian* $^{-/-}$[1] and *Rian* $^{-/-}$[2]) and in hybrid E9.5 embryos, determined by methylation-sensitive qPCR. Bars represent means ± SD from three experiments.

**(B)** RNA accumulation of the *Meg3, Rian and Mirg* ncRNAs (RT-qPCR) relative to two housekeeping genes (*β-actin* and *Gapdh*), in control, *Rian* $^{-/-}$[1] and *Rian* $^{-/-}$[2] mESCs. Bars represent means ± SD from three experiments (ns, non-significant; **** p<0.0001).

**(C)** RNA-seq on nuclear RNA extracted from Rian-/- [1] ESCs. Reads were normalised through genomic spiking. No RNA signal is detected along the *Rian* gene.

**(D)** mRNA amounts of the neural marker genes *Nestin*, *Fabp7* and *Emx1* in control, *Rian* $^{-/-}$[1] and *Rian* $^{-/-}$[2] mESCs and derived NPCs at d12 of neural differentiation. Error bars represent the ± SD of three biological replicates.

## ------------------------------------ Supplementary Figure S5 ------------------------------------

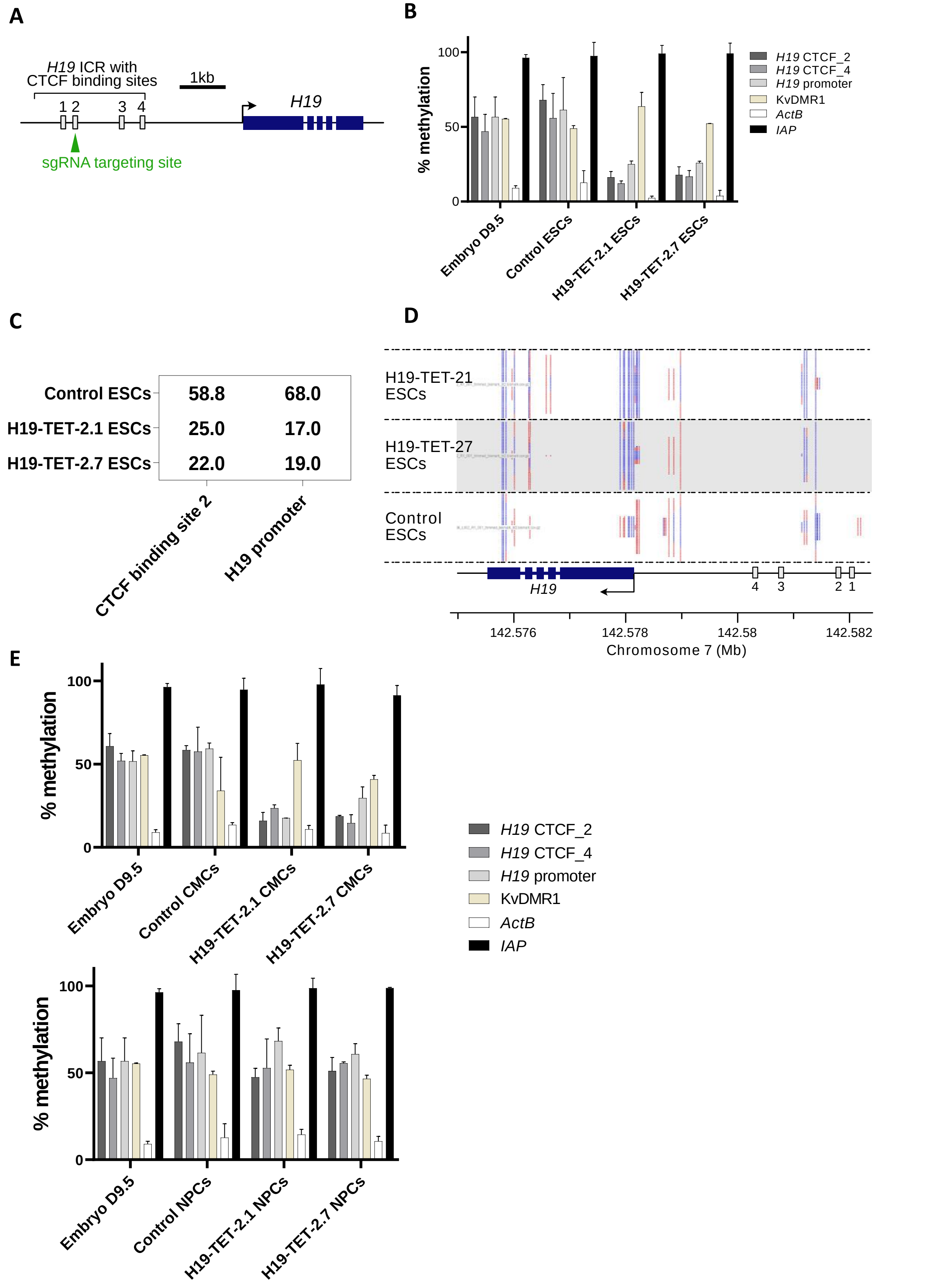

**Supplementary Figure S5. Methylation analysis of ESC lines obtained following CRISPR-dCas9-SunTag-TET1 demethylation of the *H1*9 ICR.**

**(A)** Schematic map of the *H19* ICR, with the position of the used gRNA directed to CTCF binding site 2.

**(B)** DNA methylation status in hybrid mESCs (control, H19-TET-2.1 and H19-TET-2.7) and in hybrid E9.5 embryos, as determined by methylation-sensitive qPCR. Bars represent means ± SD from three experiments.

**(C)** DNA methylation status in hybrid mESCs (control, H19-TET-2.1 and H19-TET-2.7) as determined by pyrosequencing. Data represent the means of the methylation levels of 3 CpG dinucleotides at the *H19* ICR (CTCF site-2) and 5 CpGs at the *H19* promoter.

**(D)** DNA methylation status determined by RRBS in hybrid mESCs (control, H19-TET-2.1 and H19-TET-2.7) at the *H19* gene and the *H19* ICR, with the 4 CTCF binding sites shown at grey rectangles. Stripes represent CpG dinucleotides covered by the RRBS that are methylated (red) or unmethylated (blue).

**(E)** DNA methylation status in hybrid mESC-derived CMCs and NPCs (control, H19-TET-2.1 and H19-TET-2.7) as well as in E9.5 embryos as determined by methylation-sensitive qPCR. Bars represent means ± SD from three experiments.

------------------------------------- **Supplementary Figure S6** -------------------------------------

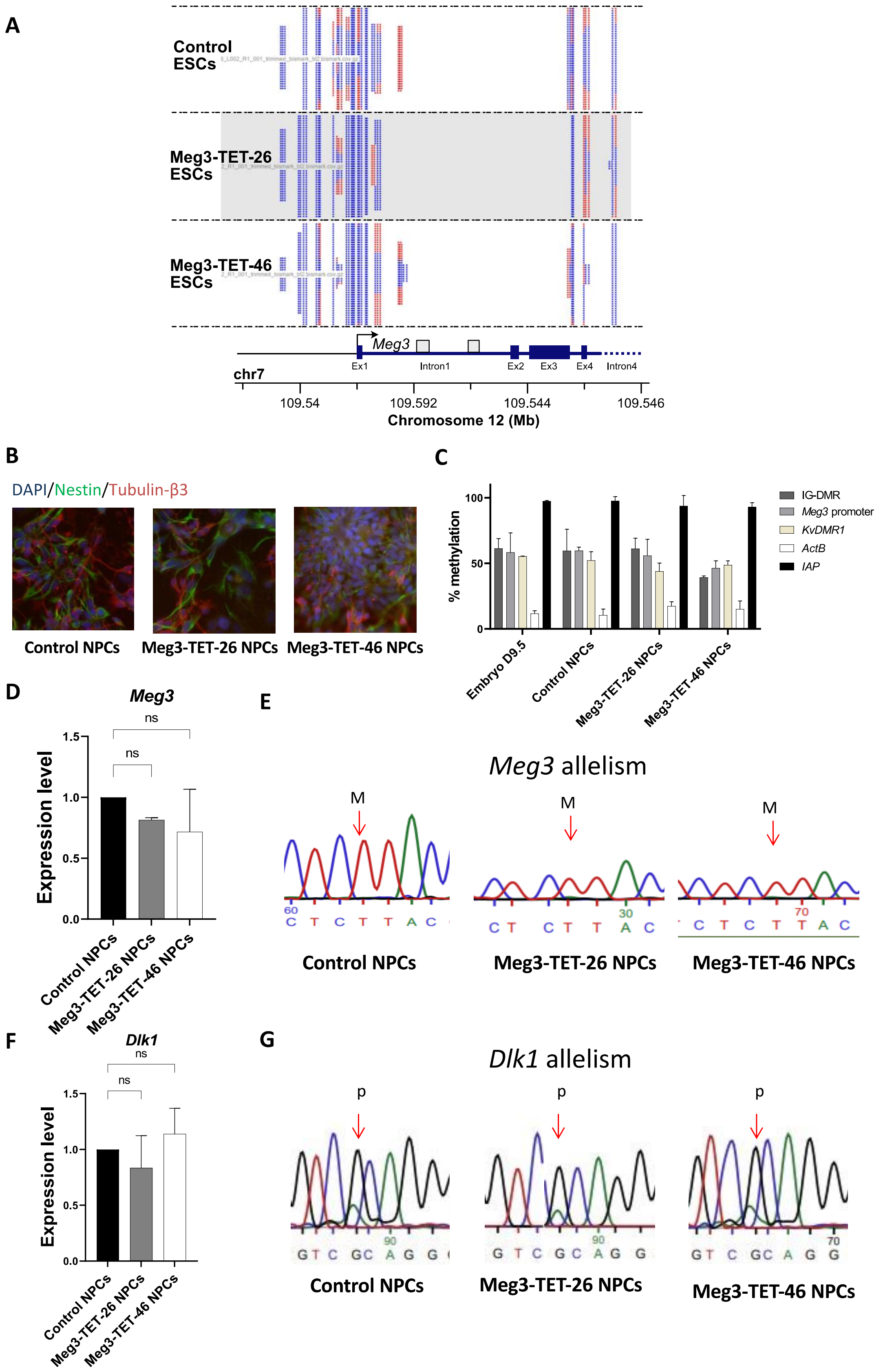

**Supplementary Figure S6. General analysis of NPCs differentiated from Meg3-TET mESCs.**

**(A)** DNA methylation status determined by RRBS in hybrid mESCs (control, Meg3-TET-26 and Meg3-TET-46) around the *Meg3* DMR. Stripes represent CpG dinucleotides covered by the RRBS that are methylated (red) or unmethylated (blue). Bottom, the *Meg3* gene is indicated, with two CTCF binding sites within intron 1 shown as grey rectangles.

**(B)** Immunofluorescence (IF) staining of Nestin (green) and Tubulin-β3 (red) with DAPI counter-staining (blue) in control, Meg3-TET-26 and Meg3-TET-46 NPCs at d12 of neural differentiation.

**(C)** DNA methylation status in hybrid mESC-derived NPCs at d12 of neural differentiation (control, Meg3-TET-26 and Meg3-TET-46) d12 and in E9.5 embryos as determined by methylation-sensitive qPCR. Bars represent means ± SD from three experiments.

**(D)** RNA accumulation of *Meg3* ncRNA (RT-qPCR) relative to housekeeping genes *β-actin* and *Gapdh* in control, Meg3-TET-26 and Meg3-TET-46 NPCs. Bars represent means ± SD from three experiments (ns, non-significant).

**(E)** Sanger sequencing-based assessment of *Meg3* expression allelism in control, Meg3-TET-26 and Meg3-TET-46 NPCs. The arrow indicates the SNP used to distinguish the parental alleles.

**(F)** *Dlk1* mRNA amounts in control, Meg3-TET-26 and Meg3-TET-46 NPCs.

**(G)** Sanger sequencing-based assessment of *Dlk1* expression allelism in control, Meg3-TET-26 and Meg3-TET-46 NPCs. The arrow indicates the SNP used to distinguish the parental alleles.

## Supplementary Figure S7

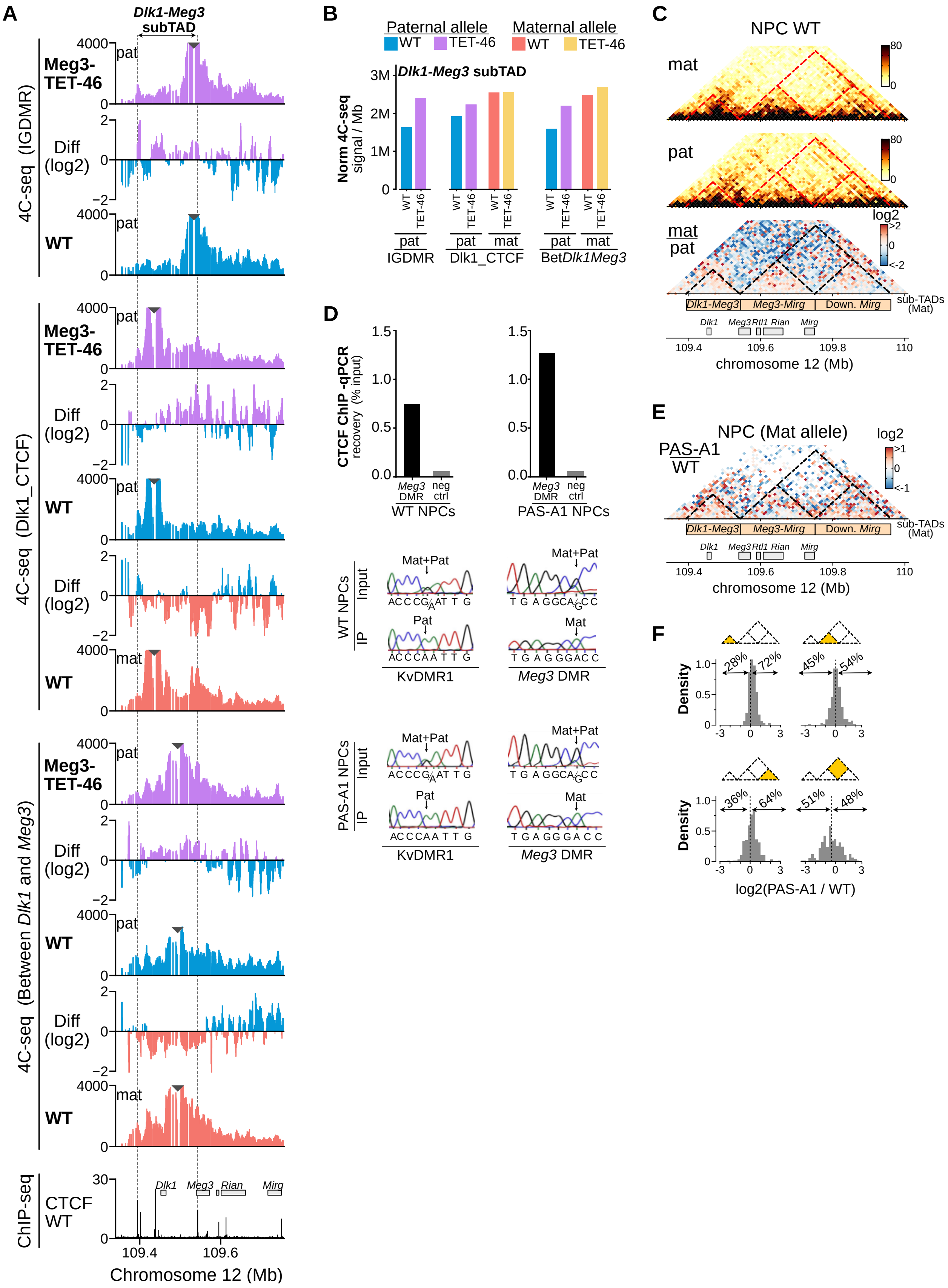

**Supplementary Figure S7. 3D chromatin organization of the *Dlk1-Dio3* domain in WT and Meg3-TET ESCs and NPCs.**

**(A)** 3D chromatin organization in hybrid mESCs (control and Meg3-TET-46) as determined by 4C-seq. Profiles are shown for the indicated alleles. Arrowheads indicate the positions of viewpoints. Ratios between tracks are indicated in-between. Tracks are aligned with published CTCF ChIP-seq profiles in WT mESCs. The maternal-specific *Dlk1-Meg3* sub-TAD (1) delineated by the CTCF peaks upstream of *Dlk1* and the maternal-specific CTCF binding at the *Meg3* DMR is highlighted in yellow.

**(B)** Quantification of normalized 4C-seq signals in the *Dlk1-Meg3* sub-TAD highlighted in (A). Quantifications are shown for the three viewpoints and, where possible, for both the parental alleles.

**(C)** 3D chromatin organization in control hybrid mESC-derived CMCs as determined by Capture Hi-C for the maternal chromosome (top) and the paternal chromosome (middle). A comparison matrix is shown below (log2 ratio). Bins are 10-kb.

**(D)** Top: CTCF ChIP on control and PAS-A1 NPCs. Percentile precipitation levels were determined by qPCR at 'binding site 2' in the *Meg3* DMR. Bottom: Sanger sequencing profiles assess allele-specificity of CTCF binding at the *Meg3* DMR and the KvDMR1.

**(E)** Comparison of the 3D chromatin organization between the maternal allele of hybrid mESC-derived control and PAS-A1 NPCs (capture-Hi-C). In the comparison matrix (log2 ratio), stronger signal in PAS-A1 NPCs is shown in red, while stronger signal in control is shown in blue. The maternal-specific sub-TADs (see Figure 6A) are outlined with dashed black lines.

**(F)** Distribution of log2 ratios between control and PAS-A1 NPCs for all the bins comprised in the 4 zones highlighted in yellow. The percentage of bins with positive/negative log2 ratio is indicated. The left-most yellow zone showed skewed log2-scores towards positive values (increased short-range interactions for the maternal allele of PAS-A1 NPCs compared to control). For the 3 other zones, the histograms are centered close to zero, indicative for an equal mix of increased and decreased interactions in PAS-A1 NPCs compared to WT NPCs for the corresponding zones on the maternal allele.

## ---------------------------------------- Supplementary Figure S8 ----------------------------------------

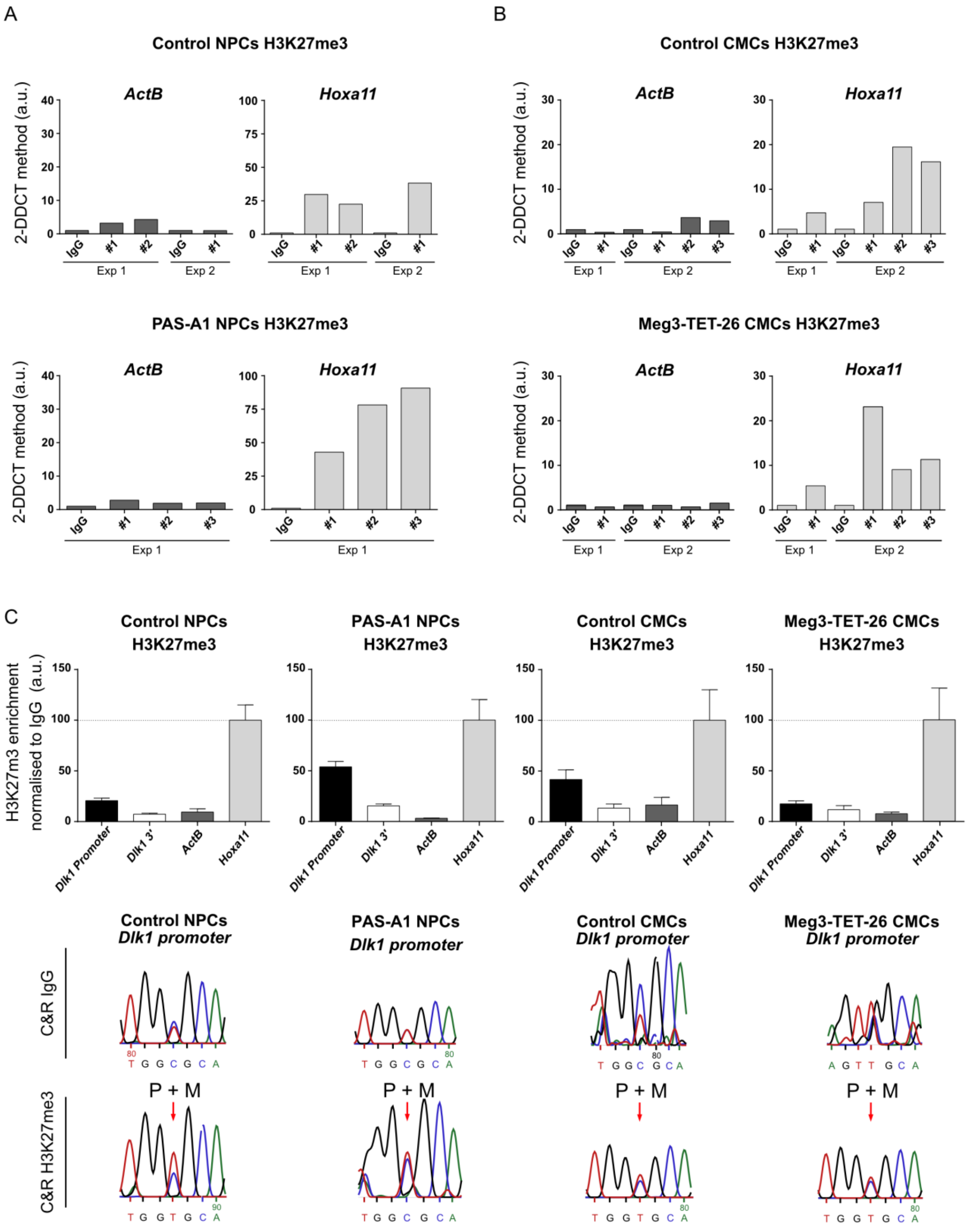

**Supplementary Figure S8. Assessment of H3K27me3 at *Dlk1* in PAS-A NPCs and Meg3-TET CMCs.**

**(A)** Relative quantification in NPCs of H3K27me3 following CUT&RUN-qPCR, based on the 2(-ΔΔCT) method (2). Experimental and/or biological replicates of control and PAS-A1 NPCs were analysed for signal enrichment at *ActB* (negative control) and *Hoxa11* (positive control), in anti-IgG (negative control) and anti-H3K27me3 CUT&RUN. #, replicate; a.u., arbitrary unit.

**(B)** Relative quantification in CMCs of H3K27me3 following CUT&RUN-qPCR, based on the 2(-ΔΔCT) method (2). Experimental and biological replicates of control and Meg3-TET-26 CMCs were tested for signal enrichment at *ActB* (negative control), *Hoxa11* (positive control), in anti-IgG (negative control) and anti-H3K27me3 CUT&RUN. #, replicate; a.u., arbitrary unit.

**(C)** Top: CUT&RUN for H3K27me3 followed by qPCR on control and PAS-A1 NPCs, and control and Meg3-TET-26 CMCs. Signal levels were determined at the *Dlk1* promoter, the *Dlk1* 3' region, *ActB* and *Hoxa11*. The *Hoxa11* signal was fixed arbitrarily at 100. Boxplots represent mean ± s.e.m. Bottom: Sanger sequencing profiles indicate the allele-specificity of H3K27me3 at the *Dlk1* promoter in the different samples.

## SUPPLEMENTARY TABLES

### Supplementary Table S1 – CRISPR tools

***<u>gRNAs used for pAS insertion and Mirg deletion:</u>***

| pAS insertion | gRNA(s) used | Homology Arm | Diagnostic PCR |
|---|---|---|---|
| *Meg3*<br>intron1<br>PAS-A lines | GGATGCTGGGGACCAGGCTA | TCTGGCTTCGGCTCCGTCCTCCTGGACATGCCGAAAGGCCAGTGCTGGGGACCTTCTCCCAAAGCCAGCCCCTTAG**AATAAAAGATCTTTATTTTCATTAGATCTGTGTGTTGGTTTTTTGTGTG**CCTGGTCCCCAGCATCCAACACGAAATTCTGCAAGGAAAAGAATCCTCAGGCACATTTTTCTCGCGGGTGTGGGG | Primers used:<br>Forward:<br>ACAGAAGACGAAGAGCTGG<br><br>Reverse:<br>CCTTGCAGAATTTCGTGTTGG<br><br>pAS insertion: 321-bp<br>WT, without pAs insertion: 272-bp |
| *Rian* deletion | gRNA1 | gRNA2 | |
| | GTCACACAGCCTTAATAAGC | GTATCTATCCCCTCTTACCT | 56.5-kb deletion |
| Scrambled gRNA (control line) | GCTTAGTTACGCGTGGACGA | | |

**<u>gRNAs used for CRISPR-dCas9-TET1 mediated DNA demethylation:</u>**

| Gene/locus | sgRNA1 | sgRNA2 | sgRNA3 |
|---|---|---|---|
| *Meg3* promoter | TCAAGATAGTCCGTCAGAAT | CTTCGTTCTTTGCTGCAGTC | GCAAGCCATCTGCCGATCCC |
| *H19* ICR | CAGATTTGGCTATAGCTAAA | | |

**Supplementary Table S2 – PCR primers for RNA analysis**

| Gene | RT-PCR primers | Sequence ID (Amplified Region) | SNPs | References |
|---|---|---|---|---|
| *Dlk1* | Fw TTGCTCCTGCTGGCTTTC<br>Rv CCTTGCAGACTCCATTGACA | NR_033813.1 (579-753) | 635, T(B6), C(JF1) | (3) |
| *Meg3* | Fw CACAGAAGACGAAGAGCTGGA<br>Rv GGTAGAGGTGCACAGCAGGT | NR_027652.1 (3-205) | 100, T(B6), A(JF1) | (3) |
| *Meg3 Exon 8* | Fw AGAAGACTGAGGACCCCAGG<br>Rv CGTGTTGTGCGTGAAGTCC | NR_027652.1 (1368-1469) | -- | This study |
| *Meg3 Intron 8* | Fw CTAGTTGAAGCCACCAAGGTAG<br>Rv CTGTGCGTTCCTGATTACCA | NC_000078.7 (109521471-109521583) | -- | This study |
| *Rian* | Fw CAATGGGTGGATCGTACCTC<br>Rv GTGCTGCCTCAGTCTTTGTG | NR_028261.1 (2546-2736) | | (3) |
| *Mirg* | Fw TCGGCAGTACATACCAGGTG<br>Rv ACTGATGGCTTCAGGTCAGG | NR_028265.1 (733-929) | | (3) |
| *Oct4* | Fw TCTTTCCACCAGGCCCCCGGCTC<br>Rv TGCGGGCGGACATGGGGAGATCC | NM_013633.3 (28-251) | | (4) |
| *Nestin* | Fw CAGCAACTGGCACACCTCAA<br>Rv CTGGTATCCCAAGGAAATGCA | NM_016701.3 (969-1131) | | (5) |
| *Emx1* | Fw GAGCGAGCCTTTGAGAAGAA<br>Rv TTGTCCTCCGATTCTGGAAC | NM_010131.2 (535-649) | | (6) |
| *Fabp7* | Fw TCCAGCTGGGAGAAGAGTTT<br>Rv CCAACCGAACCACAGACTTA | NM_021272.3 (280-348) | | (7) |
| *Gata4* | Fw GCTTCCGTTTTCTGGTTTGA<br>Rv CTGTGCCAACTGCCAGACTA | NM_0013106 10.1 119 bp | | This study |
| *Mesp1* | Fw GAGGGACTGGGCTCCATTT<br>Rv GCCTACCCTAGACCCCGAGT | NM_008588.2 190 bp | | This study |
| *Nkx2.5* | Fw GGCTTGTCCAGCTCCACT<br>Rv CATTTTACCCGGGAGCCTAC | NM_008700.2 104 bp | | (8) |
| *Tbx5* | AATCCCCAGCACAAACTCC<br>Fw<br>Rv TCTTTGGAATCAGGCTCCAG | NM_011537.3 201 bp | | This study |
| *ActB* | Fw GGCCCAGAGCAAGAGAGGTATCC<br>Rv ACGCACGATTTCCCTCTCAGC | NM_007393.5 (250-709)) | | (9) |
| *Gapdh* | Fw CGTCCCGTAGACAAAATGGT<br>Rv TGACTGTGCCGTTGAATTTG | NM_008084.3 (226-396) | | (3) |

**Supplementary Table S3 – ChIP and CUT&RUN qPCR primers**

| Gene/ locus | ChIP primers | Amplified Region | SNPs | References |
|---|---|---|---|---|
| *Dlk1 promoter* | Fw TAACCCTAAGCCCTGCACTG<br>Rv CAACGACACCCCTCTTGAAC | chr12:110692060-110692209 | 110692101, A(B6), G(JF1) | (3) |
| *Hoxa11* | Fw AGGAGAAGGGGTTCCTTCAA<br>Rv CTCCGCGGTTTGTCAATAAT | chr6:52196524-52196649 | | (10) |
| *ActB* | Fw TTCGCTCTCTCGTGGCTAGT<br>Rv GACCCTAGTGTGTCCCCAAG | chr5:143668084-143668301 | | (10) |
| KvDMR1 | Fw CGGATCACTTGAGCACTAC<br>Rv GCCAAGTGGATCGCGCCAAG | chr7:150481890-150482128 | 150481965, A(B6), G(JF1) | (11) |
| *Meg3* | Fw CATGTGGCCTTGATCCCTAC<br>Rv CACTAGGCCCCATGCTTCTA | chr12:110809733-110809897 | 110809820, G(B6), T(JF1) | (3) |
| *Meg3* DMR (CTCF site2) | Fw CCCCCTCCTACGTCAGTCTA<br>Rv AGGTCACAAGTGTTAGCTGTGTG | chr12:109508822-109508841 | 109542983, C(JF1), T(B6) | (1) |
| *H19* ICR | Fw CATGCTTAGTGGGGTCTGCA<br>Rv GCCATCAGCGCTATTGTGTG | chr7:142135336-142135355 | | (1) |
| Telomeric of *H19* (negative control) | Fw CGCATGGCACCAGAGAAGTA<br>Rv TCAGCCCCAAACAGAATCCC(3) | chr7:142185972-142185991 | | (1) |

| Genes/locus | CUT&RUN primers | Amplified region | SNPs | Reference |
|---|---|---|---|---|
| *Dlk1 promoter* | Fw TAACCCTAAGCCCTGCACTG<br>Rv CAACGACACCCCTCTTGAAC | chr12:110692060-110692209 | 110692101, A(B6), G(JF1) | (2) |
| *Dlk1 3'* | Fw ACCAAAACACTTCTTGACTCCA<br>Rv AAACCTTTCGGATCCCAGGA | chr12:109461139-109461210 | 110699380, C(B6), T(JF1) | This study |
| *Hoxa11* | Fw TGTCAATAATCCGCGCTGTC<br>Rv AGCAGAAAGATACAGGGAAGGT | chr6:52196534-52196604 | | This study |
| *ActB* | Fw TTCGCTCTCTCGTGGCTAGT<br>Rv GTGTCCGTTCTGAGTGATCC | chr5:143668242-143668301 | | This study |

## Supplementary Table S4 – Primers for quantitative DNA methylation analysis

| Gene/ locus | DNA methylation analysis | | References |
|---|---|---|---|
| IG-DMR | Fw<br>Rv | CGGTATAGGCCAAGTGGTTTGTAGC<br>CTGTTCCGCGAGTCACCCGG | (10) |
| *Meg3* promoter | Fw<br>Rv | GGGTAGGCAGAGCAGCCGGA<br>AGGGGTACCCAGCAACCCGG | (12) |
| *H19* ICR (CTCF site2) | Fw<br>Rv | CCGTTTTAGGACTGCGATGT<br>GGGTCACAAATGCCACTAGG | This study |
| *H19* ICR (CTCF site4) | Fw<br>Rv | CGTCTGCCGAGCAATATGTA<br>CAGATTTGGCTATAGCTAAA | This study |
| *H19* promoter | Fw<br>Rv | CCAGCAGCTCCCCTTTATC<br>GTTGAAGGACTGAGGGGCTA | This study |
| KvDMR1 | Fw<br>Rv | CTCAGTTCCACGATACCCTTCC<br>CTTACAGAAGCAGGGGTGGTCT | (13) |
| *ActB* | Fw<br>Rv | GGCTTTGGCTATTGCTA<br>CCTCTGGGTGTGGATGTCA | (3) |
| IAP | Fw<br>Rv | CAAATTAAAGAGCTTGCCGAGT<br>TAGGGAGAGCGGCTTTTACA | (3) |
| *Col1a2* | Fw<br>Rv | AAAGAGAAGGATTGGTCAGAGCAGT<br>GCCAAGGGAGGAGACTTAGTTG | (13) |
| *Col9a2* | Fw<br>Rv | CTCTGGACTTATTTTTATTGGGTATCTTTT<br>CAGGGAAGATGGATGTTTAAATACTG | (13) |

**Supplementary Table S5 – PCR primers for pyrosequencing analysis.**

| **Gene/ locus** | **Pyrosequencing primers** | | | **Refer ence** |
|---|---|---|---|---|
| | *Forward primer* | *Reverse primer* | *Sequencing primer* | |
| IG-DMR-5' | AATAGGTTTTTATTGGG TTTTGAGG | CACTTAACTTTACTACTA CACATCC **(Biotinylated)** | Fw TAGTTATAGAT AATAAAGT | This study |
| IG-DMR-3' | GTTATGGATTGGTGTTA AGGTT (**Biotinylated**) | CATTCCCTATACTCAAA ACATTCTC | Rv AAAACAACAT AACATAA | This study |
| *Meg3* promoter | TTTTTTAAAGTGTGGGG AATTAGTT | ACAACCTATACACCAAA AACCTTAC **(Biotinylated)** | Fw TTAGGGGGTA TAGTTG | This study |
| *Meg3* DMR (site2) | GTTTTAGGTGGTTGGGT TATTG **(Biotinylated)** | TACAAATAAATTAATTA ACAAATCACAAAT | Rv ACCAAAAACC ATTTACACA | This study |
| *H19* ICR (site2) | TGGTTTTATGAAGTTTA TGATTATGG (**Biotinylated**) | ACAAATACCACTAAAAA AACAAAACAC | Rv ATCCTTTATAT ATAAAAACC | This study |
| *H19* promoter | TTTTGTTTAAGGGATTTT AAAGTGG | CTATCAACCAATCAATA CATAACCC **(Biotinylated)** | Fw TTTGGAGAATT TTAGGA | This study |

**Supplementary Table S6 – Primers for 4C-seq analysis**

| Locus | 4C-seq primers | | SNPs | Reference |
|---|---|---|---|---|
| IG-DMR | Fw | AATGATACGGCGACCACCGAGATCTACACTCTTTCCCTACACGACGCTCTTCCGATCTtcttctatcagccctaagaatcctga | Targeting JF1 allele | (1) |
| | Rv | CAAGCAGAAGACGGCATACGAGATCGTGATGTGACTGGAGTTCAGACGTGTGCTCTTCCGATCTataaccctgcggaatgggtg | | |
| *Dlk1* distal CTCF | Fw | AATGATACGGCGACCACCGAGATCTACACTCTTTCCCTACACGACGCTCTTCCGATCTactttcacacagactctgcg | G (JF1) and A (Bl6) 4 nucleotides 3’ of the 4C forward primer. | This study |
| | Rv | CAAGCAGAAGACGGCATACGAGATCGTGATGTGACTGGAGTTCAGACGTGTGCTCTTCCGATCTgactgtccttcacacatcgg | | |
| Intergenic *Dlk1 - Meg3* | Fw | AATGATACGGCGACCACCGAGATCTACACTCTTTCCCTACACGACGCTCTTCCGATCTgcagtcccgggtcaatgtt | C (JF1) and T (Bl6) 4 nucleotides 3’ of the 4C forward primer. | This study |
| | Rv | CAAGCAGAAGACGGCATACGAGATCGTGATGTGACTGGAGTTCAGACGTGTGCTCTTCCGATCTggataggcctcaagatttccc | | |

## SUPPLEMENTARY REFERENCES


1. Lleres, D., Moindrot, B., Pathak, R., Piras, V., Matelot, M., Pignard, B., Marchand, A., Poncelet, M., Perrin, A., Tellier, V. *et al.* (2019) CTCF modulates allele-specific sub-TAD organization and imprinted gene activity at the mouse Dlk1-Dio3 and Igf2-H19 domains. *Genome Biol*, **20**, 272.
2. Livak, K.J. and Schmittgen, T.D. (2001) Analysis of relative gene expression data using real-time quantitative PCR and the 2(-Delta Delta C(T)) Method. *Methods*, **25**, 402-408.
3. Sanli, I., Lalevee, S., Cammisa, M., Perrin, A., Rage, F., Lleres, D., Riccio, A., Bertrand, E. and Feil, R. (2018) Meg3 Non-coding RNA Expression Controls Imprinting by Preventing Transcriptional Upregulation in cis. *Cell Rep*, **23**, 337-348.
4. Takahashi, K., Okita, K., Nakagawa, M. and Yamanaka, S. (2007) Induction of pluripotent stem cells from fibroblast cultures. *Nat Protoc*, **2**, 3081-3089.
5. Bondue, A., Lapouge, G., Paulissen, C., Semeraro, C., Iacovino, M., Kyba, M. and Blanpain, C. (2008) Mesp1 acts as a master regulator of multipotent cardiovascular progenitor specification. *Cell Stem Cell*, **3**, 69-84.
6. Bouschet, T., Dubois, E., Reynes, C., Kota, S.K., Rialle, S., Maupetit-Mehouas, S., Pezet, M., Le Digarcher, A., Nidelet, S., Demolombe, V. *et al.* (2016) In Vitro Corticogenesis from Embryonic Stem Cells Recapitulates the In Vivo Epigenetic Control of Imprinted Gene Expression. *Cereb Cortex*, **27**, 2418-2433.
7. Suter, D.M., Tirefort, D., Julien, S. and Krause, K.H. (2009) A Sox1 to Pax6 switch drives neuroectoderm to radial glia progression during differentiation of mouse embryonic stem cells. *Stem Cells*, **27**, 49-58.
8. Prados, B., Gomez-Apinaniz, P., Papoutsi, T., Luxan, G., Zaffran, S., Perez-Pomares, J.M. and de la Pompa, J.L. (2018) Myocardial Bmp2 gain causes ectopic EMT and promotes cardiomyocyte proliferation and immaturity. *Cell Death Dis*, **9**, 399.
9. Leeb, M., Pasini, D., Novatchkova, M., Jaritz, M., Helin, K. and Wutz, A. (2010) Polycomb complexes act redundantly to repress genomic repeats and genes. *Genes Dev*, **24**, 265-276.
10. Kota, S.K., Lleres, D., Bouschet, T., Hirasawa, R., Marchand, A., Begon-Pescia, C., Sanli, I., Arnaud, P., Journot, L., Girardot, M. *et al.* (2014) ICR Noncoding RNA Expression Controls Imprinting and DNA Replication at the Dlk1-Dio3 Domain. *Dev Cell*, **31**, 19-33.
11. Henckel, A., Chebli, K., Kota, S.K., Arnaud, P. and Feil, R. (2012) Transcription and histone methylation changes correlate with imprint acquisition in male germ cells. *Embo J*, **31**, 606-615.
12. Delaval, K., Govin, J., Cerqueira, F., Rousseaux, S., Khochbin, S. and Feil, R. (2007) Differential histone modifications mark mouse imprinting control regions during spermatogenesis. *Embo J*, **26**, 720-729.
13. Varrault, A., Eckardt, S., Girard, B., Le Digarcher, A., Sassetti, I., Meusnier, C., Ripoll, C., Badalyan, A., Bertaso, F., McLaughlin, K.J. *et al.* (2017) Mouse Parthenogenetic Embryonic Stem Cells with Biparental-Like Expression of Imprinted Genes Generate Cortical-Like Neurons That Integrate into the Injured Adult Cerebral Cortex. *Stem Cells*, **36**, 192-205.
